# Inter‐observer agreement for the histological diagnosis of invasive lobular breast carcinoma

**DOI:** 10.1002/cjp2.253

**Published:** 2021-12-10

**Authors:** Matthias Christgen, Leonie Donata Kandt, Wiebke Antonopoulos, Stephan Bartels, Mieke R Van Bockstal, Martin Bredt, Maria Jose Brito, Henriette Christgen, Cecile Colpaert, Bálint Cserni, Gábor Cserni, Maximilian E Daemmrich, Raihanatou Danebrock, Franceska Dedeurwaerdere, Carolien HM van Deurzen, Ramona Erber, Christine Fathke, Henning Feist, Maryse Fiche, Claudia Aura Gonzalez, Natalie D ter Hoeve, Loes Kooreman, Till Krech, Glen Kristiansen, Janina Kulka, Florian Laenger, Marcel Lafos, Ulrich Lehmann, Maria Dolores Martin‐Martinez, Sophie Mueller, Enrico Pelz, Mieke Raap, Alberto Ravarino, Tanja Reineke‐Plaass, Nora Schaumann, Anne‐Marie Schelfhout, Maxim De Schepper, Jerome Schlue, Koen Van de Vijver, Wim Waelput, Axel Wellmann, Monika Graeser, Oleg Gluz, Sherko Kuemmel, Ulrike Nitz, Nadia Harbeck, Christine Desmedt, Giuseppe Floris, Patrick WB Derksen, Paul J van Diest, Anne Vincent‐Salomon, Hans Kreipe

**Affiliations:** ^1^ Institute of Pathology Hannover Medical School Hannover Germany; ^2^ Institute of Pathology University Clinics Heidelberg Heidelberg Germany; ^3^ Department of Pathology Cliniques Universitaires Saint‐Luc Brussels Belgium; ^4^ Pathology and Breast Unit Champalimaud Foundation Lisbon Portugal; ^5^ Department of Pathology Universitair Ziekenhuis Leuven Leuven Belgium; ^6^ TNG Technology Consulting GmbH Budapest Hungary; ^7^ Department of Pathology University of Szeged Szeged Hungary; ^8^ Group Practice for Pathology Schweinfurt Schweinfurt Germany; ^9^ Institute of Pathology Johannes Wesling Clinics Minden Minden Germany; ^10^ Laboratorium voor pathologie AZ Delta Roeselare Belgium; ^11^ Department of Pathology Erasmus MC Cancer Institute Rotterdam The Netherlands; ^12^ Institute of Pathology, University Hospital Erlangen Friedrich‐Alexander‐Universität Erlangen‐Nürnberg (FAU), and Comprehensive Cancer Center Erlangen‐EMN (CCC ER‐EMN) Erlangen Germany; ^13^ Institute of Pathology Martin‐Luther‐University Halle‐Wittenberg Halle (Saale) Germany; ^14^ Institute of Pathology Diakonissenkrankenhaus Flensburg Flensburg Germany; ^15^ Institute of Pathology Aurigen Aurigen SA Lausanne Switzerland; ^16^ Conway Institute of Biomolecular and Biomedical Research University College Dublin Dublin Ireland; ^17^ Department of Pathology University Medical Center Utrecht Utrecht The Netherlands; ^18^ Institute of Pathology and GROW – School for Oncology and Developmental Biology Maastricht University Medical Center Maastricht The Netherlands; ^19^ Institute of Pathology University Clinics Hamburg‐Eppendorf Hamburg Germany; ^20^ Germany and Pathocom Network for Pathology Osnabrück Germany; ^21^ Institute of Pathology University Hospital Bonn Bonn Germany; ^22^ 2nd Department of Pathology Semmelweis University Budapest Budapest Hungary; ^23^ Institut de Pathologie et Genetique Gosselies Gosselies Belgium; ^24^ Institute of Pathology Viersen Viersen Germany; ^25^ Institute of Pathology University of Cagliari Cagliari Italy; ^26^ Department of Pathology OLV Ziekenhuis Aalst Aalst Belgium; ^27^ Department of Pathology University Hospitals Leuven, Campus Gasthuisberg Leuven Belgium; ^28^ Cancer Research Institute Ghent Ghent University Hospital Ghent Belgium; ^29^ Department of Pathology UZ Brussel, Vrije Universiteit Brussel Brussels Belgium; ^30^ Institute of Pathology Celle Celle Germany; ^31^ West German Study Group Moenchengladbach Germany; ^32^ Ev. Hospital Bethesda Breast Center Niederrhein Moenchengladbach Germany; ^33^ Gynecologic University Clinic Hamburg‐Eppendorf (UKE) Hamburg Germany; ^34^ Breast Unit, Kliniken Essen‐Mitte, Essen, Germany and Charité ‐ Universitätsmedizin Berlin, Department of Gynecology with Breast Center Berlin Germany; ^35^ Department of Gynecology and Obstetrics, Breast Center University of Munich (LMU) and CCCLMU Munich Germany; ^36^ Laboratory for Translational Breast Cancer Research, Department of Oncology KU Leuven Leuven Belgium; ^37^ Department of Imaging and Radiology, Laboratory for Cell and Tissue Translational Research KU‐Leuven/UZ Leuven Leuven Belgium; ^38^ Pathology‐Genetics‐Immunology Department, Institut Curie PSL Research University Paris France

**Keywords:** lobular breast carcinoma, diagnosis, quality assurance, beta‐catenin, p120‐catenin, tubular elements, ELBCC/LOBSTERPOT consortium

## Abstract

Invasive lobular breast carcinoma (ILC) is the second most common breast carcinoma (BC) subtype and is mainly driven by loss of E‐cadherin expression. Correct classification of BC as ILC is important for patient treatment. This study assessed the degree of agreement among pathologists for the diagnosis of ILC. Two sets of hormone receptor (HR)‐positive/HER2‐negative BCs were independently reviewed by participating pathologists. In set A (61 cases), participants were provided with hematoxylin/eosin (HE)‐stained sections. In set B (62 cases), participants were provided with HE‐stained sections and E‐cadherin immunohistochemistry (IHC). Tumor characteristics were balanced. Participants classified specimens as non‐lobular BC versus mixed BC versus ILC. Pairwise inter‐observer agreement and agreement with a pre‐defined reference diagnosis were determined with Cohen's kappa statistics. Subtype calls were correlated with molecular features, including *CDH1*/E‐cadherin mutation status. Thirty‐five pathologists completed both sets, providing 4,305 subtype calls. Pairwise inter‐observer agreement was moderate in set A (median *κ* = 0.58, interquartile range [IQR]: 0.48–0.66) and substantial in set B (median *κ* = 0.75, IQR: 0.56–0.86, *p* < 0.001). Agreement with the reference diagnosis was substantial in set A (median *κ* = 0.67, IQR: 0.57–0.75) and almost perfect in set B (median *κ* = 0.86, IQR: 0.73–0.93, *p* < 0.001). The median frequency of *CDH1*/E‐cadherin mutations in specimens classified as ILC was 65% in set A (IQR: 56–72%) and 73% in set B (IQR: 65–75%, *p* < 0.001). Cases with variable subtype calls included E‐cadherin‐positive ILCs harboring *CDH1* missense mutations, and E‐cadherin‐negative ILCs with tubular elements and focal P‐cadherin expression. ILCs with trabecular growth pattern were often misclassified as non‐lobular BC in set A but not in set B. In conclusion, subtyping of BC as ILC achieves almost perfect agreement with a pre‐defined reference standard, if assessment is supported by E‐cadherin IHC. *CDH1* missense mutations associated with preserved E‐cadherin protein expression, E‐ to P‐cadherin switching in ILC with tubular elements, and trabecular ILC were identified as potential sources of discordant classification.

## Introduction

Invasive lobular breast carcinoma (ILC) is the second most common histological breast carcinoma (BC) subtype [1, 2, 3]. ILC accounts for approximately 15% of all BC cases and is defined by distinct histomorphological characteristics reviewed previously [2].

ILC is a special type of BC, in terms of both tumor biology and clinical behavior [4, 5]. ILC is hormone receptor (HR)‐positive/HER2‐negative (i.e. luminal) in the vast majority of cases and is mainly driven by inactivation of the *CDH1/*E‐cadherin cell adhesion molecule [6, 7, 8, 9]. ILC shows a distinct landscape of mutational alterations (e.g. different frequencies of mutations in *ARID1A*, *CDH1*, *ERBB2, GATA3*, *TP53*, and other cancer‐related genes) [8, 10]. In addition, ILC is over‐represented in metastatic BC and is associated with distinct metastatic sites [11, 12]. The prognostic impact of molecular profiling assays, such as the Oncotype DX RS score and the PAM50 ROR score, which aid in clinical decisions for or against adjuvant chemotherapy, differs between ILC and non‐lobular BC [13, 14, 15, 16]. Regarding Oncotype DX, ILC is associated with a three‐fold lower prevalence of high‐risk RS scores, but the 5‐year disease‐free survival is similar in patients with lobular and non‐lobular BC [13, 15]. Regarding PAM50, the 10‐year distant recurrence rates for patients with intermediate risk ROR scores are nearly twice as high for ILC as for BC of no special type (NST, 18 versus 10%) [16]. Hence, correct classification of BC as ILC is thought to be prerequisite for adequate interpretation of prognostic profiling assays [14, 15, 16]. Moreover, correct classification of ILC is relevant for MRI indication [17, 18].

Recently, exploratory subgroup analyses from two large clinical BC trials have indicated suboptimal accuracy of the histopathological diagnosis of ILC. In the MINDACT trial, central histology review confirmed ILC in only 60% (395/654) of BC cases classified as ILC by local assessment (Cohen's *κ* approximately 0.68) [19]. In the WSG PlanB trial, central histology review confirmed ILC in 66% (253/385) of BC cases classified as ILC by local assessment (Cohen's *κ* = 0.70) [15]. However, assessment of BC subtypes was not the primary study aim of these clinical trials.

Few morphological studies have ever determined the agreement among pathologists for the diagnosis of ILC [20, 21, 22]. These studies were conducted up to 30 years ago and did not include ancillary immunohistochemistry (IHC) for E‐cadherin [20, 21, 22]. The aim of the present study was to assess the degree of agreement among pathologists for the diagnosis of ILC (with and without E‐cadherin IHC). In addition, this study sought to identify potential sources of discordant subtype calls.

## Materials and methods

### Tumor specimens

Tumor specimens included 123 formalin‐fixed paraffin‐embedded (FFPE) core needle biopsies (CNBs) with invasive, HR‐positive/HER2‐negative early BC (Table 1). Tumor specimens were from patients enrolled in the West German Study Group (WSG) ADAPT trial (NCT01779206) [23, 24]. Eighty‐one BCs of NST (according to central pathology review within the ADAPT trial) and 42 ILCs (according to central pathology review within ADAPT) were randomly selected from the ADAPT Trial Translational Research Registry (German Cancer Aid, grant 70112954) based on study IDs. Tumor characteristics are provided in Table 1 and in supplementary material, Tables S1–S4. FFPE tissue blocks and histological sections, which had originally been prepared for central pathology review in the ADAPT trial, were retrieved from the central tumor bank at the Hannover Medical School. Next, BCs of NST were spiked with ILCs at a ratio of approximately 2:1. Case order was arbitrary. All specimens were anonymized. This study was approved by the local ethics committee (MHH, Hannover, Germany, reference number 2716‐2015).

**Table 1:** Tumor characteristics, as defined by the reference standard, are balanced between set A and set B.

		All cases		Set A		Set B			
	Reference	*n*	%	*n*	%	*n*	%	Test	*P* value
All cases		123	100	61	50	62	50		
Subtype	NST	81	100	40	49	41	51	FET	0.948
	ILC	42	100	21	50	21	50		
Grade	G1	3	100	1	33	2	67	CSTT	0.316
	G2	79	100	43	54	36	46		
	G3	41	100	17	41	24	59		
mBSR: architecture	1	0	100	0	0	0	0	CSTT	0.325
	2	22	100	13	59	9	41		
	3	101	100	48	48	53	52		
mBSR: nuc. grade	1	7	100	3	43	4	57	CSTT	0.786
	2	73	100	38	52	35	48		
	3	43	100	20	47	23	53		
mBSR: proliferation	1	22	100	13	59	9	41	CSTT	0.239
	2	77	100	38	49	39	51		
	3	24	100	10	42	14	58		
ER	Neg.	1	100	1	100	0	0	FET	0.492
	Pos.	121	100	59	49	62	51		
	N.A.	1	100	1	100	0	0		
PR	Neg.	9	100	5	56	4	44	FET	0.743
	Pos.	114	100	56	49	58	51		
HER2	0/1+	101	100	48	48	53	52	CSTT	0.247
	2+/F.‐neg.	20	100	13	65	7	35		
	2+/F. N.A.	1	100	0	0	1	100		
	3+, 2+/F.‐pos.*	1	100	0	0	1	0		
Ki67	<10%	6	100	3	50	3	50	CSTT	0.929
	10–19%	37	100	17	46	20	54		
	20–34%	70	100	37	53	33	47		
	35–100%	10	100	4	40	6	60		
E‐cadherin	Neg.	41	100	20	49	21	51	FET	1.000
	Aberrant	0	100	0	0	0	0		
	Pos.	82	100	41	50	41	50		
*CDH1* status	Wild‐type	90	100	45	50	45	50	FET	1.000
	Mutant	33	100	16	48	17	52		
Beta‐catenin	Neg.	37	100	18	49	19	51	CSTT	0.950
	Focally pos.	4	100	2	50	2	50		
	Pos.	82	100	41	50	41	50		
p120‐catenin	Neg.	12	100	7	58	5	42	FET	0.559
	Pos.	111	100	54	49	57	51		
p120‐catenin mislocation	Membranous	76	100	40	53	36	47	FET	0.228
	Mislocated	35	100	14	40	21	60		
	Not informative	12	100	7	58	5	42		
P‐cadherin	Neg.	102	100	53	52	49	48	FET	0.338
	Focally pos.	21	100	8	38	13	62		
	Pos.	0	100	0	0	0	0		

CSTT, chi‐square test for trends (set A *versus* set B); ER, estrogen receptor; F., HER2 fluorescence in situ hybridization (FISH); FET, Fisher's exact test (set A versus set B); mBSR, modified Bloom–Scarf–Richardson Score grading components; neg., negative; pos., positive; PR, progesterone receptor.

* Corresponds to one case with a minor HER2‐pos. Subclone, <5% of cells.

### Reference diagnosis

Specimens were pre‐annotated with (1) histological BC subtype according to local pathologies within the ADAPT trial (documented in 2012–2016); (2) histological BC subtype according to central pathology review within the ADAPT trial (diagnosis made by a team of 2–4 expert breast pathologists headed by Prof. HK, based on hematoxylin/eosin [HE]‐stained sections, aided by E‐cadherin IHC [clone ECH‐6, Zytomed, Berlin, Germany] for all cases, documented in 2012–2016); (3) additional characteristics including histological grade (modified Bloom–Scarff–Richardson score), grading score components, histological variants (when applicable), estrogen receptor, progesterone receptor, and HER2 status, Ki67 index, and E‐cadherin status (according to central IHC and central review within ADAPT, documented in 2012–2016); (4) molecular features including beta‐catenin, p120‐catenin, and P‐cadherin expression (central IHC, documented in 2018–2019); and (5) *CDH1*/E‐cadherin mutation status (according to central next‐generation sequencing (NGS), documented in 2018–2020). Tumor characteristics according to central pathology review within the ADAPT trial served as a pre‐defined reference standard in this study.

### Immunohistochemistry

IHC was carried out in the central pathology unit of the ADAPT trial, using a Benchmark Ultra automated stainer (Ventana, Tucson, AZ, USA). Central IHC scoring was carried out by a team of 2–4 expert pathologists on a multi‐headed microscope. Immunological reagents and IHC scoring methods are summarized in the supplementary material, Table S1.

### Next‐generation sequencing

Genomic DNA was extracted as described previously [25]. The *CDH1*/E‐cadherin mutation status was determined by NGS using a customized *CDH1* NGS panel and the Ion S5 system (Life Technologies, Carlsbad, CA, USA). This NGS panel covered the complete protein‐coding sequence of the *CDH1* gene, the 5′‐untranslated region (UTR) sequence of exon 1, and the 3′‐UTR sequence of exon 16, as described previously [25, 26]. Variant annotation was performed with ANNOVAR software and database tools (http://www.openbioinformatics.org/annovar) [27]. Due to limited tumor tissue in CNBs, matched FFPE resection specimens corresponding to the CNBs were used for mutational analysis.

### Slide sets

For virtual microscopy, histological sections were scanned using a dotSlide scanner microscope (Olympus GmbH, Münster, Germany). Slides were divided into two sets for re‐assessment based on HE‐stained sections (set A, *n* = 61 cases) and for re‐assessment based on HE‐stained sections and E‐cadherin IHC (set B, *n* = 62 cases). Tumor characteristics were balanced (Table 1).

### Participants

Participants included 35 experienced board‐certified pathologists from 27 institutions from nine countries (Belgium, France, Germany, Hungary, Ireland, Italy, The Netherlands, Portugal, and Switzerland). The approximate geographical distribution of participants is illustrated in supplementary material, Figure S1. Of the 35 pathologists, 28 (80%) were from academic institutions or university clinics and had special interest in BC. Of the 35 participants, 7 (20%) were general pathologists from non‐academic institutions, and all were involved in BC diagnostics on a regular basis. Three pathologists who had been involved in the ADAPT trial central review (in 2012–2016) participated in the re‐assessment in 2019–2020 (participant IDs p01_i01, p02_i01, and p03_i01). For histological re‐assessment, all participants independently interpreted all cases from slide sets A and B. In 2019, participants could opt for glass slides or virtual microscopy. In 2020–2021, participants could only opt for virtual microscopy, which was related to restrictions during the coronavirus pandemic. Using checkmark matrices, participants classified each specimen as BC of NST/non‐lobular BC versus mixed BC (NST or other non‐lobular BCs mixed with an ILC component) versus ILC. In set B, ancillary E‐cadherin IHC stainings were classified as positive versus aberrant (such as nuclear mislocalization or fragmented staining) versus negative. Participants were instructed to make their classification calls as they would usually do in routine diagnostics. Written text comments were optional. On the discretion of individual participants, strongly reduced E‐cadherin immunoreactivity was occasionally classified as an aberrant E‐cadherin status, and written text comments were provided in such instances. All participants were blinded to the reference standard and NGS results.

### Statistics

For two‐dimensional presentation of BC subtype calls, specimens and participants were clustered using single linkage and ClustVis software [28]. For assessment of inter‐observer agreement, pairwise Cohen's *κ* values (ranging from −1 to 1) for the exact BC subtype (NST/non‐lobular BC versus mixed BC versus ILC) were calculated for each pair of pathologists (*n* = 595 pairs) using JMP software (JMP 11, SAS Institute Corporation, Cary, NC, USA). For assessment of agreement with the reference standard, Cohen's *κ* values were also calculated based on each participant's subtype calls and the reference standard using VassarStats [29]. Interquartile range (IQR) was calculated with GraphPad Prism 5 (GraphPad Software, Inc., San Diego, CA, USA). Cohen's *κ* values were interpreted as follows: <0.0 (poor agreement), 0.0–0.20 (slight agreement), 0.21–0.40 (fair agreement), 0.41–0.60 (moderate agreement), 0.61–0.80 (substantial agreement), and 0.81–1.00 (almost perfect agreement) [30]. The Wilcoxon test was used to assess statistical significance of different median *κ* values obtained in sets A and B. Accuracy was calculated as the proportion of cases concordantly classified as NST/non‐lobular BC versus mixed BC/ILC by participants and the reference (accuracy for the detection of a lobular tumor component). Statistical significance of different proportional participant calls for ILC in ILC subsets with different growth pattern (dissociated, single files, and trabecular) was determined with the Kruskal–Wallis test.

## Results

### Baseline characteristics

Two sets of histological slides (sets A and B) were compiled from HR‐positive/HER2‐negative BCs. Tumors were randomly selected from patients enrolled in the WSG ADAPT trial (NCT01779206) [23, 24]. Specimens were pre‐annotated with BC subtypes according to local pathologies and according to central pathology review in the ADAPT trial (based on expert assessment and aided by upfront E‐cadherin IHC for all cases). BC subtypes and E‐cadherin status according to central review served as a pre‐defined reference standard. Tumor characteristics were balanced between sets A and set B (Table 1).

ILCs, as defined by the reference standard, associated with loss of E‐cadherin expression, *CDH1*/E‐cadherin mutation, loss of beta‐catenin expression, and aberrant cytosolic/nuclear p120‐catenin (all *p* < 0.001) (supplementary material, Table S4). In detail, loss of E‐cadherin, as defined by the reference IHC status, was evident in 40/42 (95%) ILCs and in 1/80 (1%) BC of NST. The *CDH1* mutation frequency was 32/42 (76%) in ILC and 1/82 (1%) in BC of NST (supplementary material, Table S4). One ILC harbored two different mutations (case B031). Most *CDH1* mutations (26/34, 76%) were frameshift or nonsense mutations generating premature stop codons (supplementary material, Figure S2). E‐cadherin expression was lost in 31/34 (91%) BCs harboring *CDH1* mutations. Conversely, E‐cadherin expression was preserved in 3/34 (9%) BCs harboring *CDH1* mutations, all of which were missense mutations (supplementary material, Figure S2). The frequency of *CDH1* mutations was balanced between sets A and B (Table 1). Regarding histological growth patterns, 33/42 (79%) ILCs were classic ILCs with predominant dissociated growth pattern or single file growth pattern. The remaining ILCs (9/42, 21%) showed trabecular or solid growth patterns and were balanced between sets A and B (supplementary material, Table S3). Rare ILC variants, such as histiocytoid ILC [reviewed in Ref. 2], were not included.

BC subtype calls by local pathologies in the ADAPT trial showed substantial agreement with the reference standard in set A (*κ* = 0.74) and in set B (*κ* = 0.71) (Table 2). This is consistent with previous findings in the WSG PlanB trial (*κ* = 0.70) [15].

**Table 2:** Agreement between local and central pathology (in the ADAPT trial).

	Central pathology (reference standard)					
	Set A			Set B		
Local pathology	NST	Mixed	ILC	NST	Mixed	ILC
NST/non‐lobular BC	38	0	4	38	0	4
Mixed BC	0	0	1	0	0	1
ILC	2	0	16	3	0	16
Cohen's kappa	0.74			0.71		
Accuracy*	90%			89%		

* Accuracy for a lobular tumor component (mixed and ILC grouped together).

### BC subtype calls

**Figure 1:**
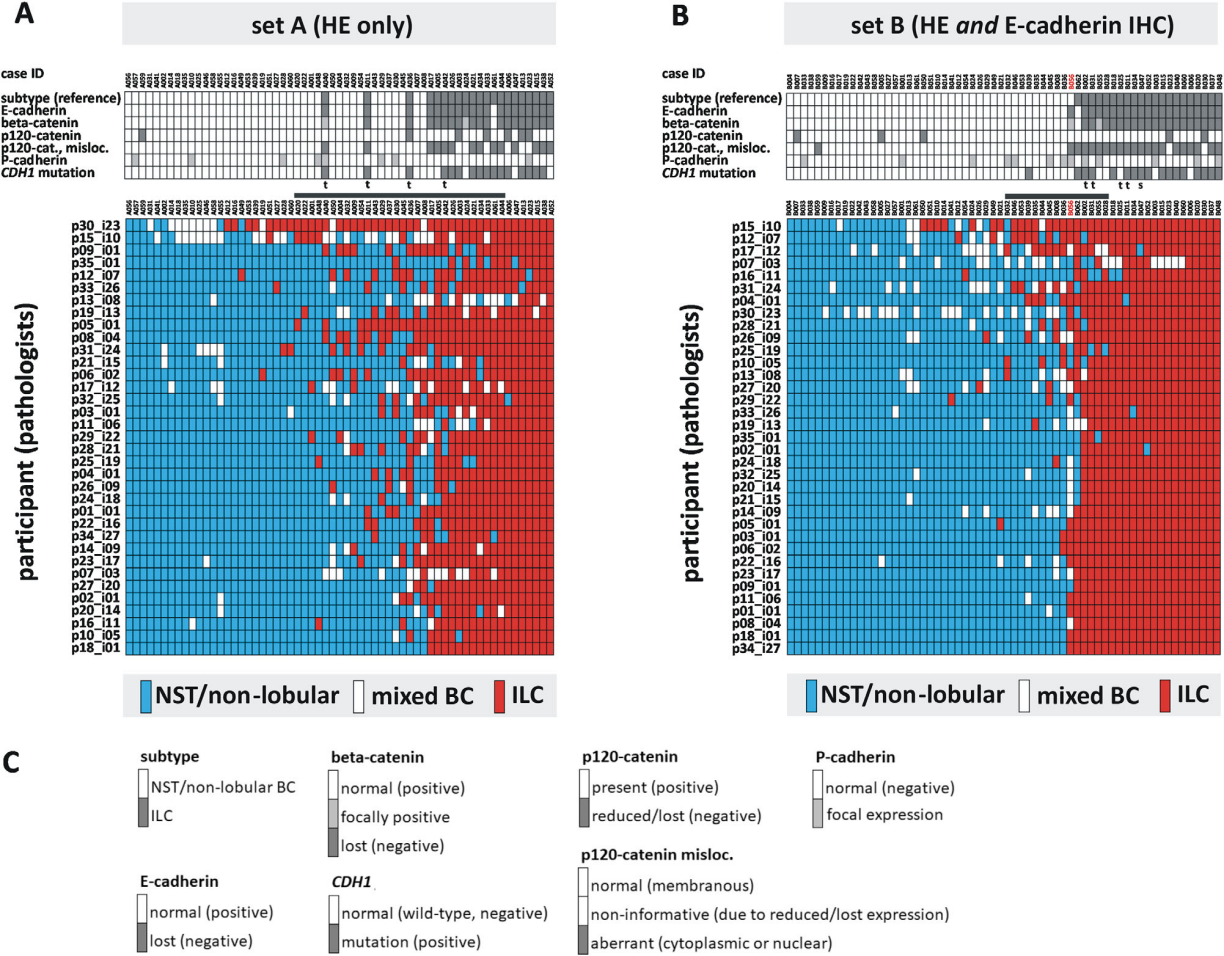
BC subtype calls. (A) Thirty‐five experienced pathologists independently classified 61 BCs based on HE‐stained sections (set A). (B) Thirty‐five pathologists classified another 62 BC specimens based on HE‐stained sections and E‐cadherin IHC (set B). Tumor characteristics, as defined by the reference standard, are shown in the top panels. A two‐dimensional presentation of subtype calls is shown in the lower panels. Each row represents the calls of one participating pathologist. Each column represents a specimen. Specimens are ordered from left to right according to increasing calls for ILC. Participants are ordered from top to bottom according to a clustering analyses (single linkage). BC subtypes are coded by color, as indicated in the legend. The horizontal gray bar indicates BC with variable subtype calls (<9 and >91% participant calls for ILC). Case B056, which received the most controversial subtype calls, is highlighted in red. ILCs with predominantly trabecular or solid growth pattern (according to the reference) are marked with a ‘t’ or ‘s’, respectively. (C) Legend for tumor characteristics, as defined by the reference standard.

**Figure 2:**
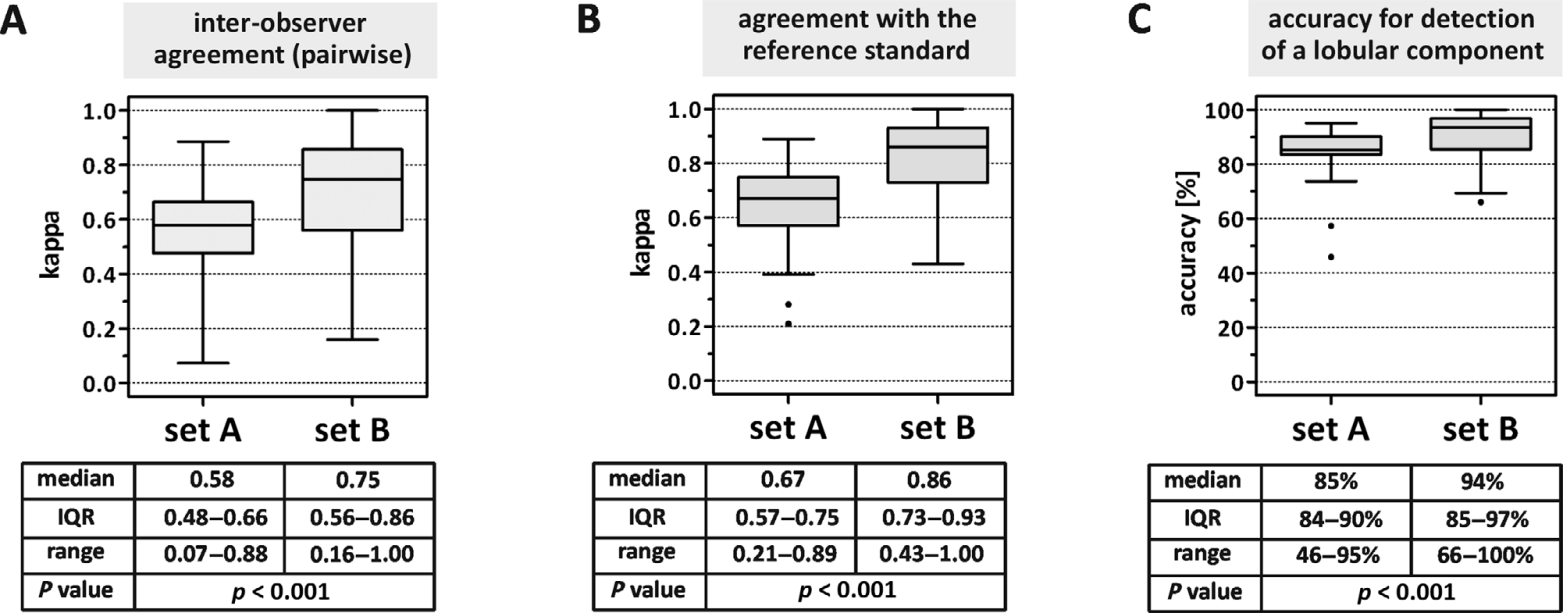
BC subtype agreement. (A) Inter‐observer agreement. BC subtypes (NST versus mixed BC versus ILC) were classified by 35 pathologists. Pairwise Cohen's *κ* values were calculated for 595 pairs of participants. (B) Agreement with the reference standard. BC subtypes (NST versus mixed BC versus ILC) were classified by 35 pathologists. Cohen's *κ* values were calculated for each pathologist's calls as compared to the reference diagnosis. (C) Agreement with the reference standard for a lobular tumor component (mixed BC and ILC grouped together) expressed as accuracy (%). Cohen's *κ* values were calculated for each pathologist's calls as compared to the reference diagnosis. Data are presented as traditional Tukey plots showing the distribution of *κ* values. Horizontal lines indicate the median, boxes indicate the IQR, and whiskers indicate the 1.5‐fold interquartile distance, or the minimal/maximal values, whichever is shorter. Significance was determined with the Wilcoxon test.

Between October 2019 and February 2021, 48 experienced board‐certified pathologists from 11 countries were invited to review slide set A (HE only) and slide set B (HE plus E‐cadherin IHC). Thirty‐five pathologists (73%) from nine countries completed both sets, providing 4,305 subtype calls (NST/non‐lobular BC versus mixed BC versus ILC) (Figure 1). Of the 35 pathologists, 28 (80%) were from academic institutions and had special interest in BC. Figure 1 shows a two‐dimensional presentation of subtype calls. An alternative data visualization, based on percent participant calls for ILC per specimen, is included in the supplementary material, Figure S3. Pairwise inter‐observer agreement was moderate in set A (median *κ* = 0.58, IQR: 0.48–0.66) and substantial in set B (median *κ* = 0.75, IQR: 0.56–0.86) (Figure 2A). Agreement with the reference standard was substantial in set A (median *κ* = 0.67, IQR: 0.57–0.75) and almost perfect in set B (median *κ* = 0.86, IQR: 0.73–0.93) (Figure 2B). The proportion of cases concordantly classified as NST/non‐lobular BC versus mixed BC/ILC by participants and the reference was also calculated (accuracy for a lobular tumor component). Median accuracy was 85% in set A (IQR: 84–90%) and 94% in set B (IQR: 85–97%) (Figure 2C). Median inter‐observer agreement, median agreement with the reference standard, and median accuracy were all significantly higher in set B compared to set A (all *p* < 0.001) (Figure 2).

### E‐cadherin status calls

In set B, participants also assessed the E‐cadherin IHC status based on centrally stained sections, providing another 2,170 IHC calls (E‐cadherin positive versus aberrant versus negative). Agreement with the reference E‐cadherin IHC status was almost perfect among participating pathologists (median *κ* = 0.96, IQR: 0.93–0.96, and median accuracy 98%, IQR: 97–98%) (supplementary material, Figure S4).

### *CDH1* mutation frequency by BC subtype

**Figure 3:**
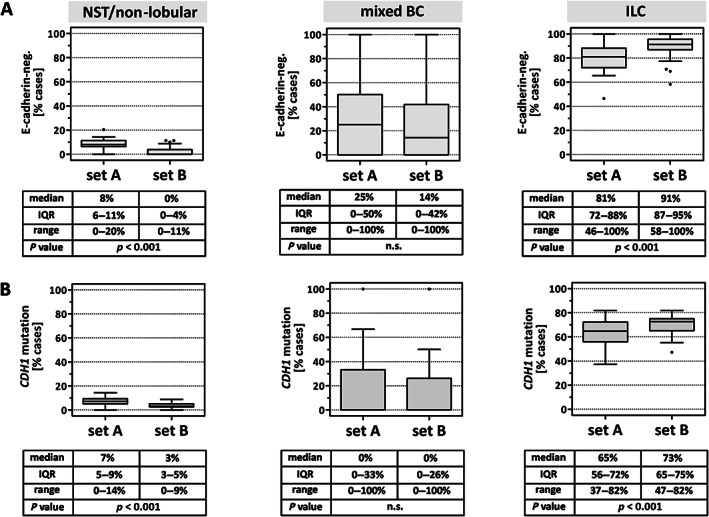
Loss of E‐cadherin and *CDH1* mutation in BC subtypes. (A) BC subtypes (NST versus mixed BC versus ILC) were classified by 35 pathologists. Shown is the proportion of cases with loss of E‐cadherin expression, as defined by the reference IHC status, in those specimens classified by participants as BC of NST (left panel), mixed BC (middle panel), or ILC (right panel). (B) Shown is the proportion of cases with a detectable *CDH1* mutation in those specimens classified by participants as BC of NST (left panel), mixed BC (middle panel), or ILC (right panel). Data are presented as traditional Tukey plots. Horizontal lines indicate the median, boxes indicate the IQR, and whiskers indicate the 1.5‐fold interquartile distance, or the minimal/maximal values, whichever is shorter. Significance was determined with the Wilcoxon test.

Next, BC subtype calls were correlated with the reference E‐cadherin IHC status and with the *CDH1* mutation status (Figure 3). On an inter‐individual basis, participants showed different frequencies for loss of E‐cadherin expression and *CDH1* mutation in BCs classified as ILC. The median frequency for loss of E‐cadherin expression in ILC was 81% in set A (IQR: 72–88%) and 91% in set B (IQR: 87–95%) (Figure 3A). The median frequency for a detectable *CDH1* mutation in ILC was 65% in set A (IQR: 56–72%) and 73% in set B (IQR: 65–75%). Hence, BCs classified as ILC by participants showed a greater proportion of *CDH1* mutations in set B compared to set A (*p* < 0.001) (Figure 3B).

### Sources of discordant subtype calls

**Figure 4:**
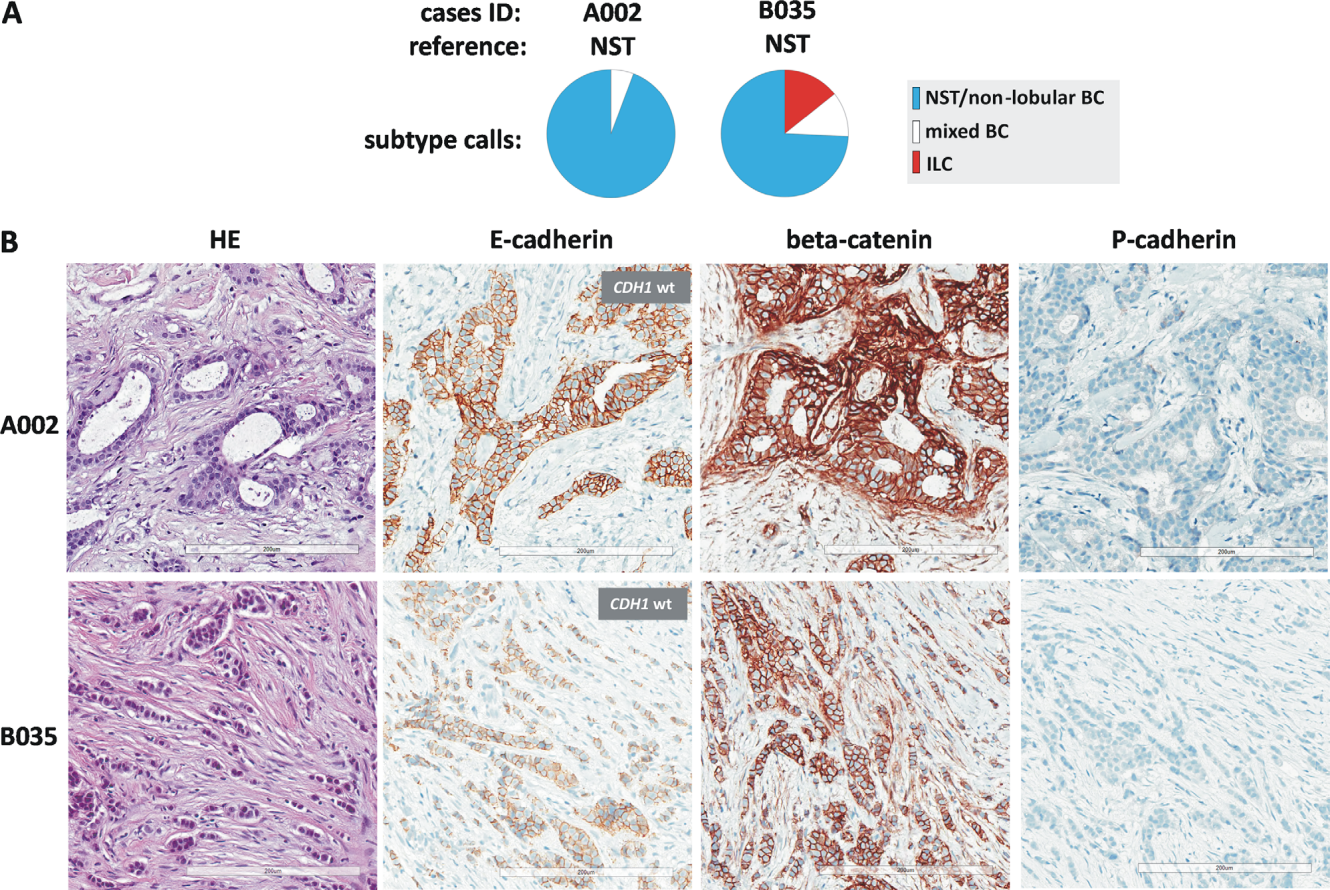
BC subtype calls in selected cases. (A) Pie charts showing proportional subtype calls for cases A002 and B035. Case IDs and BC subtypes according to the reference standard are given on top. (B) Representative photomicrographs of HE‐stained sections (left) at ×200 magnification. Scale bars correspond to 200 μm. Photomicrographs of IHC stainings for E‐cadherin, beta‐catenin, and P‐cadherin on consecutive serial sections are also provided (right). Insets fitted over E‐cadherin IHC stainings indicate the *CDH1* mutation status, as determined by NGS.

**Figure 5:**
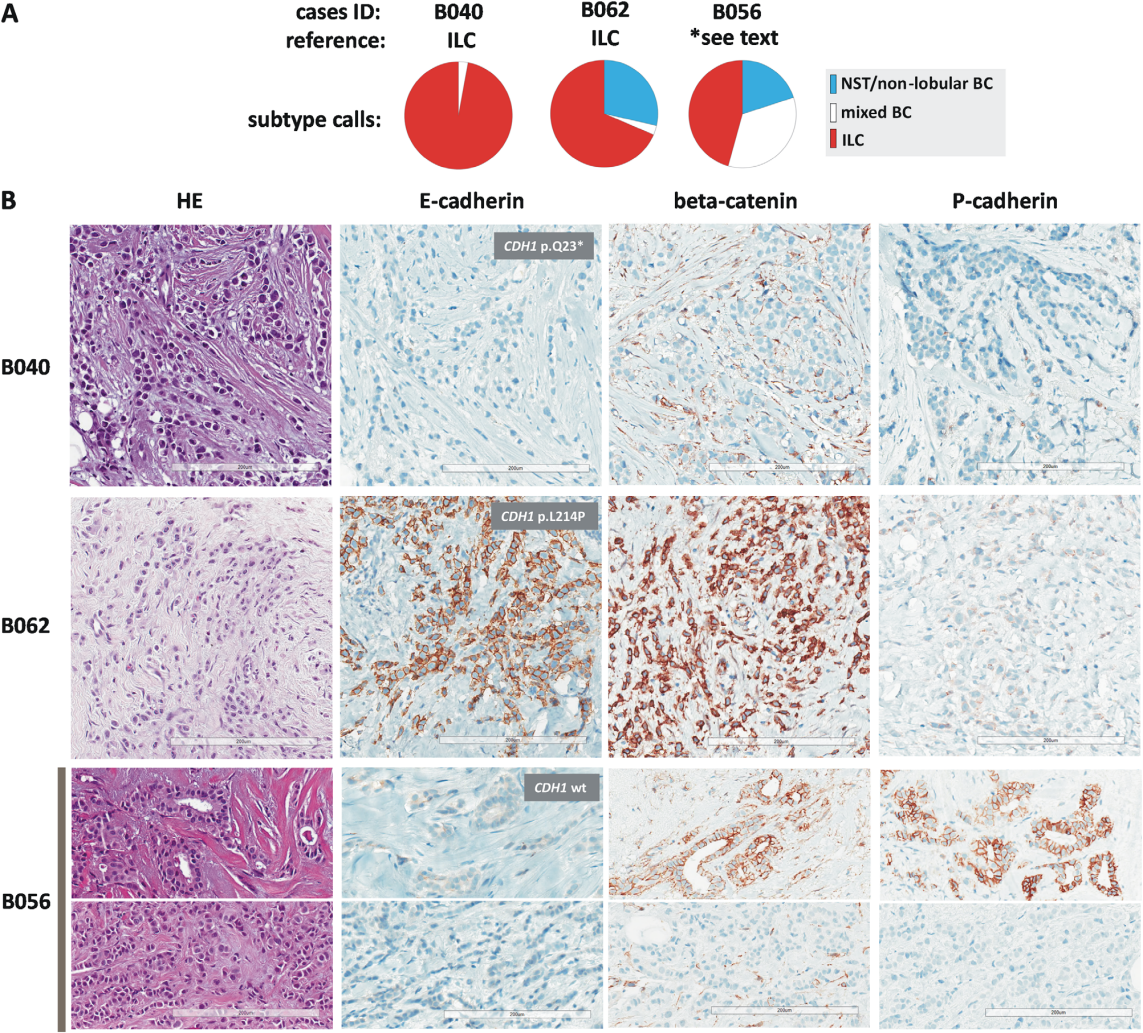
BC subtype calls in selected cases. (A) Pie charts showing proportional subtype calls for cases B040, B062, and B056. Case IDs and BC subtypes according to the reference standard are given on top. (B) Representative photomicrographs of HE‐stained sections (left) at ×200 magnification. Scale bars correspond to 200 μm. Photomicrographs of IHC stainings for E‐cadherin, beta‐catenin, and P‐cadherin are also provided (right). Insets fitted over E‐cadherin IHC stains indicate the *CDH1* mutation status, as determined by NGS. For case B056, the upper photomicrograph shows an area with tubular elements and the lower photomicrographs shows an area with non‐cohesive tumor cells arranged in single files. Note E‐cadherin to P‐cadherin switching in tubular elements of cases B056 (lower right).

To identify potential sources of discordant subtyping, specimens were categorized (based on subtype calls of participants) into three groups corresponding to: (I) consistent classification as non‐lobular BC, (II) variable classification, and (III) consistent classification as ILC. Cutoffs were set at <9 and >91% participant calls for ILC. Using these stringent cutoffs, category‐II specimens (variable classification) accounted for 30/61 (49%) cases in set A and for 15/62 (24%) cases in set B (*p* = 0.005) (supplementary material, Table S5). Figures 4 and 5 illustrate representative specimens. Cases A002 and B040 are representative for specimens that were consistently classified as NST and ILC, respectively. These cases featured growth in ductal structures or dissociated single cells, and were E‐cadherin‐positive or ‐negative, respectively. Cases B035, B062, and B056 are representative for specimens that received variable subtype calls (category‐II specimens) (Figures 4 and 5).

Case B035 was classified as BC of NST, according to the reference standard. Case B035 featured rather cohesive tumor cells arranged in slender trabeculae and round aggregates. There was no lobular carcinoma *in situ* (LCIS). E‐cadherin immunoreactivity was positive, but the staining intensity appeared slightly reduced. This may have been the reason for classification as ILC by 5/35 (14%) participants. Loss of E‐cadherin is typically accompanied by loss of beta‐catenin and cytosolic translocation of p120‐catenin in ILC [31, 32, 33]. Beta‐catenin binds to the cytoplasmic region of E‐cadherin and links adherens junctions (AJs) to the actin cytoskeleton via alpha‐catenin. Downregulation of beta‐catenin reflects the disassembly of AJs [31, 32]. Additional IHC stainings (which were not provided to participants for subtyping) showed strong, membranous beta‐catenin immunoreactivity (Figure 4B). p120‐catenin showed membranous immunoreactivity as well (not shown). NGS revealed a wild‐type *CDH1* sequence. Cases similar to B035 (E‐cadherin‐positive, *CDH1* wild‐type, beta‐catenin‐positive, and p120‐catenin membranous) accounted for 16/30 (53%) category‐II cases in set A, and for 8/15 (53%) category‐II cases in set B.

Case B062 was classified as an E‐cadherin‐positive ILC, according to the reference standard. Case B062 featured medium‐sized tumor cells and a dissociated growth pattern. Foci of LCIS (E‐cadherin‐negative) were present (supplementary material, Figure S5). However, E‐cadherin IHC showed strong, but focally incomplete or fragmented membranous immunoreactivity in ILC cells (Figure 5B). The E‐cadherin IHC status was classified as positive by 32/35 (91%) participants, and as aberrant/negative by 3/35 (9%) participants (supplementary material, Figure S4A). Immunoreactivity for E‐cadherin may have been the reason for classification as BC of NST by 10/35 (29%) participants. Additional IHC stainings demonstrated membranous beta‐catenin immunoreactivity, but partially cytosolic p120‐catenin. NGS revealed a *CDH1* missense mutation (p.L214P), which provided an explanation for the preserved E‐cadherin immunoreactivity, as observed with the ECH‐6 antibody [34]. To date, anti‐E‐cadherin antibody clones other than ECH‐6 have not been tested on this case. Repeated *CDH1* sequencing of microdissected sub‐regions (with and without LCIS) confirmed the presence of the p.L214P mutation in the invasive tumor component (data not shown). Specimens similar to B062 (lobular morphology, *CDH1* missense mutation) accounted for 1/30 (3%) category‐II cases in set A, and for 3/15 (20%) category‐II cases in set B.

Case B056 was initially classified as BC of NST in the CNB, according to the reference standard. However, the final reference diagnosis was changed to ILC after a repeated evaluation of the CNB together with the corresponding resection specimen, which was obtained 4 weeks later. Case B056 featured non‐cohesive tumor cells arranged in single files and cohesive tumor cells arranged in tubules (Figure 5B). Single files accounted for approximately 80–85% and tubules for approximately 15–20% of the invasive carcinoma. Both growth patterns were intimately mixed (supplementary material, Figure S6). Foci of LCIS were present (LCIS with pagetoid extension in mammary ducts, E‐cadherin‐negative) (supplementary material, Figure S5). E‐cadherin immunoreactivity was also lost in the invasive tumor cells. Tubules were E‐cadherin‐negative too. Adjacent normal mammary ducts showed strong E‐cadherin immunoreactivity, which verified appropriate IHC staining (not shown). Case B056 was classified as ILC, mixed BC, or NST/non‐lobular BC by 16/35 (46%), 12/35 (34%), and 7/35 (20%) participants. Strikingly, 17 participants provided written comments to case B056. Three participants (9%) also considered tubulo‐lobular BC as a possible diagnosis. However, tubulo‐lobular BC is described as E‐cadherin‐positive in the literature [2, 35, 36, 37]. Written comments provided by the participants are documented in the supplementary material, Table S6, and illustrate the difficulties associated with the interpretation of case B056. NGS showed a wild‐type *CDH1* sequence. Additional IHC stainings revealed strong expression of the alternate cell adhesion molecule P‐cadherin in the tubules (Figure 5B). P‐cadherin can function as a partial substitute for E‐cadherin and can partially rescue AJ formation in the absence of E‐cadherin [26, 38]. Consistently, case B056 showed membranous beta‐catenin immunoreactivity in the tubules, but not in dissociated tumor cells (Figure 5B). This peculiar phenotype (lobular growth pattern mixed with tubules, E‐cadherin‐negative, and P‐cadherin focally positive) has recently been identified as a variant of ILC termed ‘ILC with tubular elements’ [26]. This ILC variant is characterized by focal E‐cadherin to P‐cadherin switching [4, 26]. Specimens with E‐ to P‐cadherin switching accounted for 1/30 (3%) category‐II cases in set A, and for 3/15 (20%) category‐II cases in set B. Focal P‐cadherin expression was significantly associated with category‐II specimens in set B (*p* = 0.015) (supplementary material, Table S7).

Taken together, potential sources of discordant subtyping among participants included E‐cadherin‐positive BC of NST with trabecular growth pattern, *CDH1* missense mutations associated with preserved E‐cadherin protein expression, and E‐ to P‐cadherin switching in ILC with tubular elements.

### ILCs classified as BC of NST in set A

Finally, we also evaluated BCs with discordant subtyping between participants and the reference. We focused on set A (assessment based on HE‐stained sections), because median agreement with the reference was lower in set A compared to set B. In fact, set A included three cases that were classified as ILC by the reference, but as NST/non‐lobular BC by most participants (Figure 1A). These cases were A040, A011, and A036, and they received 28/35 (80%), 27/35 (77%), and 19/35 (54%) participant calls for NST, respectively (supplementary material, Figure S7A). All three cases featured growth in broad ribbons and/or ragged clusters and lacked LCIS (supplementary material, Figure S7B). Typical ILC features were scanty and included only focal intracytoplasmic vacuoles and focal nuclear compression (reviewed in [2]). All three cases were E‐cadherin‐negative and harbored *CDH1* frameshift mutations (supplementary material, Figure S7B). According to the reference, these cases were described as ILC with predominantly trabecular growth pattern. Of note, set A and set B included four ILCs with predominantly trabecular growth pattern each (Figure 1). In set A, ILCs with trabecular growth received significantly fewer participant calls for ILC compared to ILCs with classic growth pattern (dissociated growth or single files) (*p* < 0.001) (supplementary material, Figure S7C). In set B, ILCs with trabecular growth received nearly as many participant calls for ILC as ILCs with classic growth pattern (supplementary material, Figure S7D). Hence, ILCs with trabecular growth pattern were often misclassified as NST/non‐lobular BC in set A, but not in set B. This implies that, without E‐cadherin IHC, the majority of ILCs with predominantly trabecular growth pattern is at risk of being misclassified as NST.

### Educational assessment of BC subtypes

For educational purposes, sets A and B were also evaluated by a small group of non‐pathologists (*n* = 18, mainly medical students after training in basic histopathology). Educational assessment results are summarized in the supplementary material, Table S8.

## Discussion

Correct histopathological classification of ILC is important for MRI indication, interpretation of prognostic profiling assays, and systemic therapy [14, 15, 16, 17, 18]. Recently, two large clinical BC trials (MINDACT trial and WSG PlanB trial) have indicated suboptimal concordance between BC subtypes determined by local versus central pathology [15, 19]. The present study assessed the magnitude of agreement among pathologists for the diagnosis of ILC in an experimental approach.

Two histological slide sets (sets A and B, one with HE‐stained sections only and the other with complementary E‐cadherin IHC) were randomly compiled from HR‐positive/HER2‐negative BCs. Tumor specimens were pre‐annotated with detailed tumor characteristics, including the best achievable histological BC subtype classification. This was based on a consensus of experts aided by upfront E‐cadherin IHC (central pathology review of the WSG ADAPT trial) [24]. Tumor characteristic and BC subtypes, as defined by central pathology review in the ADAPT trial, served as a pre‐defined reference standard. Tumor characteristics were balanced between sets A and B. Accordingly, subtype calls from sets A and B were appropriate for comparative statistical analyses. Comparison of local versus central BC subtype calls from the ADAPT trial showed substantial agreement in both sets A and B (*κ* = 0.74 and *κ* = 0.71, respectively). This is consistent with previous findings from the WSG PlanB trial (*κ* = 0.70) [15].

Thirty‐five experienced pathologists reviewed both slide sets and classified each specimen as NST/non‐lobular BC versus mixed BC versus ILC. Participating pathologists achieved substantial agreement with the reference in set A (median *κ* = 0.67, based on HE‐stained sections), but almost perfect agreement with the reference in set B (median *κ* = 0.86, based on HE‐stained sections and E‐cadherin IHC). Hence, BC subtyping can achieve almost perfect agreement with a pre‐defined reference standard, if assessment is supported by E‐cadherin IHC.

Irrespective of the reference standard, which may also not always represent a biological truth, subtyping supported by E‐cadherin IHC showed significantly improved pairwise inter‐observer agreement. Moreover, subtyping supported by E‐cadherin IHC resulted in a significantly increased proportion of *CDH1* mutations in those BCs that were classified as ILC. Furthermore, assessment of the E‐cadherin IHC status *per se* showed almost perfect pairwise inter‐observer agreement (median *κ* = 0.89) and achieved an excellent accuracy (median accuracy 98%).

The role of E‐cadherin IHC for BC subtyping in routine diagnostics is rather undefined [39]. One expert panel has advocated that E‐cadherin IHC should probably not be performed in cases considered as ILC on HE‐stained sections [40]. Another work has argued that E‐cadherin IHC is of limited value, because as much as 23% of *CDH1*‐mutant BCs showed preserved E‐cadherin expression [41]. This finding was not confirmed in the present study (3/34, 9%, in the present work). Collectively, the clearly improved consistency in BC subtyping and the increased frequency of *CDH1* mutations in those BCs that were classified as ILC, as shown in the present study, may serve as strong arguments to recommend E‐cadherin as an obligate IHC marker to be assessed in every newly diagnosed BC. Currently, health insurances may not always cover the extra costs for E‐cadherin IHC, which may be one factor that detains pathologists from ordering this key staining on a regular basis. IHC for beta‐catenin or p120‐catenin may be useful as non‐obligate secondary stainings to objectify ambiguous cases with either uncertain E‐cadherin status or discrepant E‐cadherin staining but lobular morphology [33]. NGS of *CDH1* and/or further genes involved in defective cell adhesion, such as *CTNNA1/*alpha‐catenin, may be an optional ancillary method, which can help to objectify the diagnosis of ILC in the case of a deleterious mutation, but cannot rule out ILC in the case of a wild‐type sequence [42, 43, 44]. Detection of aberrant *CDH1* promoter methylation may be of limited or no value as an ancillary diagnostic method. The relevance of epigenetic silencing of *CDH1* in ILC is controversial [10, 44, 45, 46].

Three earlier studies have determined the inter‐observer agreement among pathologists for the histological diagnosis of ILC [20, 21, 22]. These studies were reported by Kiaer *et al*, Cserni, and Longacre *et al* [20, 21, 22]. These studies were conducted either with fewer ILC specimens, or with fewer pathologists and did not include E‐cadherin IHC [20, 21, 22]. Inter‐observer agreement was highly variable in these earlier studies (Cohen's *κ* = 0.74, *κ* = 0.31, and *κ* = 0.80, respectively) [20, 21, 22]. BC subtype assignment by artificial intelligence‐based image analysis has been reported to achieve similar agreement (*κ* = 0.66) [47].

Potential limitations of the present study include (1) use of virtual microscopy, (2) centrally but not locally stained E‐cadherin IHC, (3) restriction of cases to HR‐positive/HER2‐negative BCs (i.e. luminal BCs), (4) omission of consensus discussions in favor of a pre‐defined reference diagnosis, (5) the increased frequency of ILCs in the study collection (34%) as compared to population‐based BC cohorts (approximately 15%), and (6) composition of slide sets A and B of BCs of NST spiked with ILCs (exclusion of BCs classified as mixed BCs as per central reference diagnosis). Recent studies have demonstrated that diagnostic accuracy in surgical pathology using virtual microscopy (whole slide imaging) is noninferior to microscopy using original glass slides [48]. Hence, virtual microscopy was considered an acceptable method for the present study. The findings of the present study are important, but validation in larger BC cohorts or clinical BC trial collections is warranted.

This study also sought to identify tumor characteristics associated with discordant subtype calls. E‐cadherin‐positive BC of NST with trabecular growth pattern, ILC with trabecular growth pattern, *CDH1* missense mutations associated with preserved E‐cadherin expression, and E‐ to P‐cadherin switching in ILC with tubular elements were identified as potential sources of discordant classification. Therefore, the diagnosis of ILCs should be made with caution, if E‐cadherin IHC is positive and no adjacent LCIS is present. These cases may require additional workup with IHC for beta‐catenin or p120‐catenin, or with DNA sequencing if the morphology is not beyond any doubt (dissociated growth pattern). Increased awareness of ILCs with tubular elements, an ILC variant associated with E‐ to P‐cadherin switching, may further improve inter‐observer agreement [2, 26]. A more widespread use of E‐cadherin and P‐cadherin IHC in the differential diagnosis of selected BC cases, namely in those with mixed‐appearing morphology, may be advantageous [26].

In summary, subtyping of BC as ILC achieves almost perfect agreement with a pre‐defined reference standard, if assessment is supported by E‐cadherin IHC. Potential sources of discordant subtype calls include E‐cadherin‐positive ILCs harboring *CDH1* missense mutations, which may display an E‐cadherin immunoreactivity pattern essentially undistinguishable from normal E‐cadherin expression. Moreover, ILCs with tubular elements due to E‐cadherin to P‐cadherin switching and ILCs with trabecular growth pattern are a potential source of discordant subtype calls or misclassification. Increased awareness of these phenomena may improve consistent classification of BC as ILC in the future.

## Author contributions statement

MC, HK, AVS, PWBD and CD designed the study. HC performed immunohistochemical analyses. MC and HK evaluated the central reference IHC. SB and UL performed mutational analyses. LDK and MC carried out statistical analyses. All authors contributed to data collection and data analysis. MC, LDK, HK, GC, AVS, GF, GC, PWBD and CD wrote the manuscript.

## Supporting information


**Figure S1.** Approximate geographical distribution of participants
**Figure S2.** *CDH1* mutations
**Figure S3.** Percent participant calls for ILC
**Figure S4.** E‐cadherin status calls and agreement
**Figure S5.** Histology of LCIS in cases B056 and B062
**Figure S6.** Histology of case B056 (ILC with tubular elements)
**Figure S7.** Histology of ILCs classified as NST in set A
**Table S1.** Antibodies and IHC scoring
**Table S2.** Balanced characteristics of BCs of NST in sets A and B
**Table S3.** Balanced characteristics of ILCs in sets A and B
**Table S4.** Characteristics associated with ILC, as defined by the reference
**Table S5.** Proportions of category‐II specimens in sets A and B
**Table S6.** Participant's written comments to case B056
**Table S7.** Characteristics associated with category‐II specimens
**Table S8.** Educational BC subtype assessment
