# Supplementary material for: Inter‐observer agreement for the histological diagnosis of invasive lobular breast carcinoma
Source: J Pathol Clin Res. 2021 Dec 10;8(2):191–205. doi: 10.1002/cjp2.253 (PMC8822373; doi:10.1002/cjp2.253)
Supplement: Supplementary file 1 — Figure S1. Approximate geographical distribution of participants Figure S2. CDH1 mutations Figure S3. Percent participant calls for ILC Figure S4. E‐cadherin status calls and agreement Figure S5. Histology of LCIS in cases B056 and B062 Figure S6. Histology of case B056 (ILC with tubular elements) Figure S7. Histology of ILCs classified as NST in set A Table S1. Antibodies and IHC scoring Table S2. Balanced characteristics of BCs of NST in sets A and B Table S3. Balanced characteristics of ILCs in sets A and B Table S4. Characteristics associated with ILC, as defined by the reference Table S5. Proportions of category‐II specimens in sets A and B Table S6. Participant's written comments to case B056 Table S7. Characteristics associated with category‐II specimens Table S8. Educational BC subtype assessment [file CJP2-8-191-s001.pdf]

Inter-observer agreement for the histological diagnosis of invasive lobular breast cancer

M Christgen *et al.* *J Pathol Clin Res* DOI: 10.1002/cjp2.253

Contents

Figure S1. Approximate geographical distribution of participants
Figure S2. *CDH1* mutations
Figure S3. Percent participant calls for ILC
Figure S4. E-cadherin status calls and agreement
Figure S5. Histology of LCIS in cases B056 and B062
Figure S6. Histology of case B056 (ILC with tubular elements)
Figure S7. Histology of ILCs classified as NST in set A

Table S1. Antibodies and IHC scoring
Table S2. Balanced characteristics of BCs of NST in set A and B
Table S3. Balanced characteristics of ILCs in set A and B
Table S4. Characteristics associated with ILC, as defined by the reference
Table S5. Proportions of category II specimens in set A and B
Table S6. Participant's written comments to case B056
Table S7. Characteristics associated with category-II specimens
Table S8. Educational BC subtype assessment

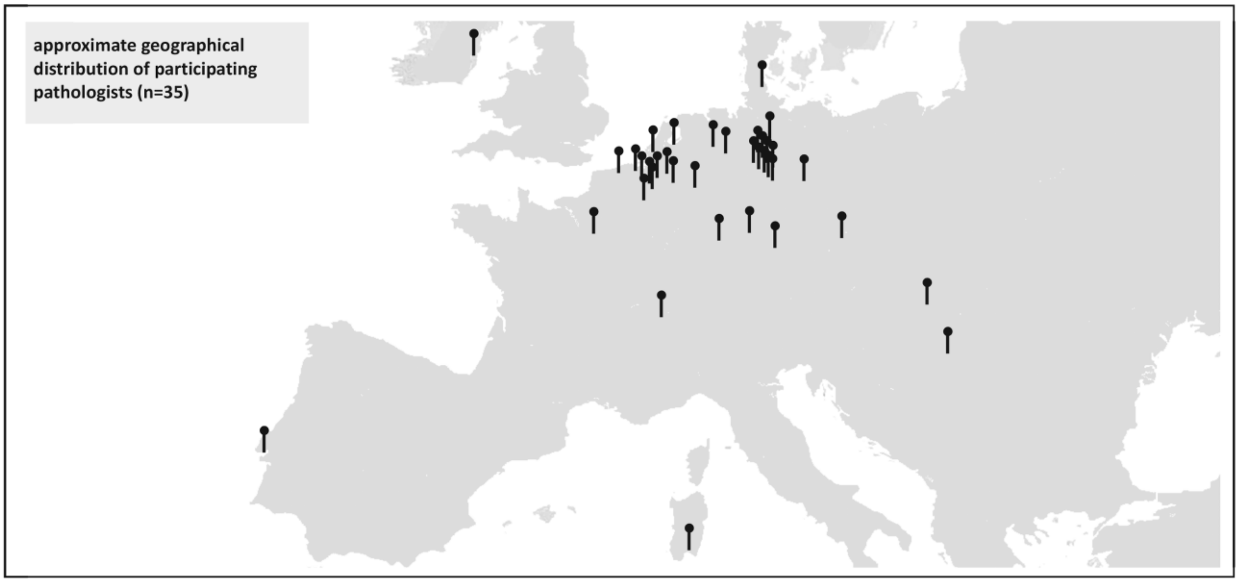

Figure S1. Approximate geographical distribution of participating pathologists. Each pin corresponds to a participant. Participants included 35 experienced board-certified pathologists from 27 institutions from 9 countries (Belgium, France, Germany, Hungary, Ireland, Italy, the Netherlands, Portugal, Switzerland).

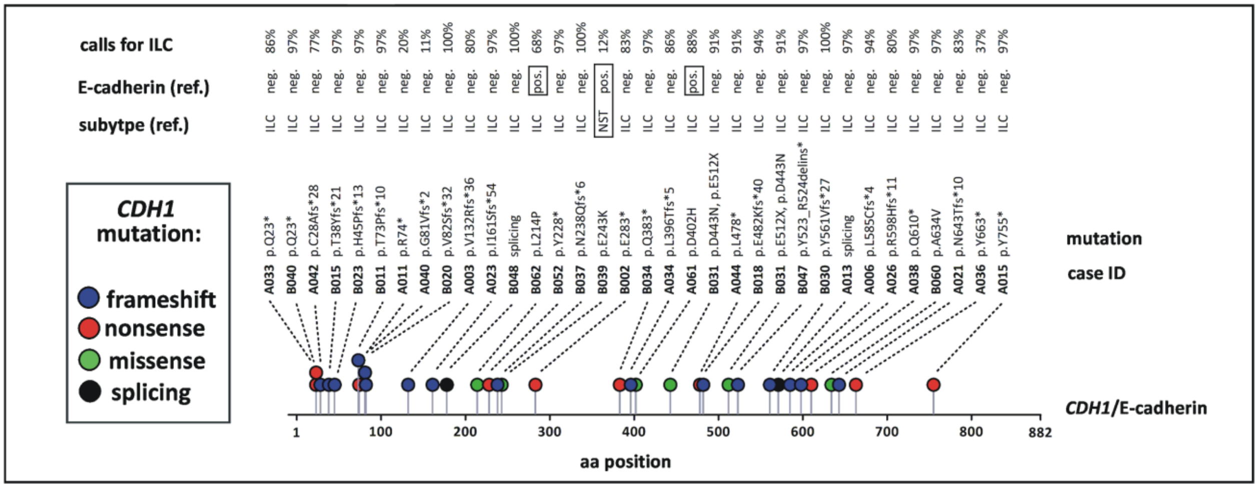

Figure S2. *CDH1* mutations. Lollipop plot of *CDH1* mutations detected in the study collection. Blue circles correspond to frameshift mutations, red circles correspond to nonsense mutations, green circles correspond to missense mutations and black circles correspond to mutations affecting splicing sites. Assignment of the corresponding BCs to either ILC or NST/non-lobular BC by participants and the reference is shown on the top. The E-cadherin protein expression status of the corresponding specimens is also shown on the top.

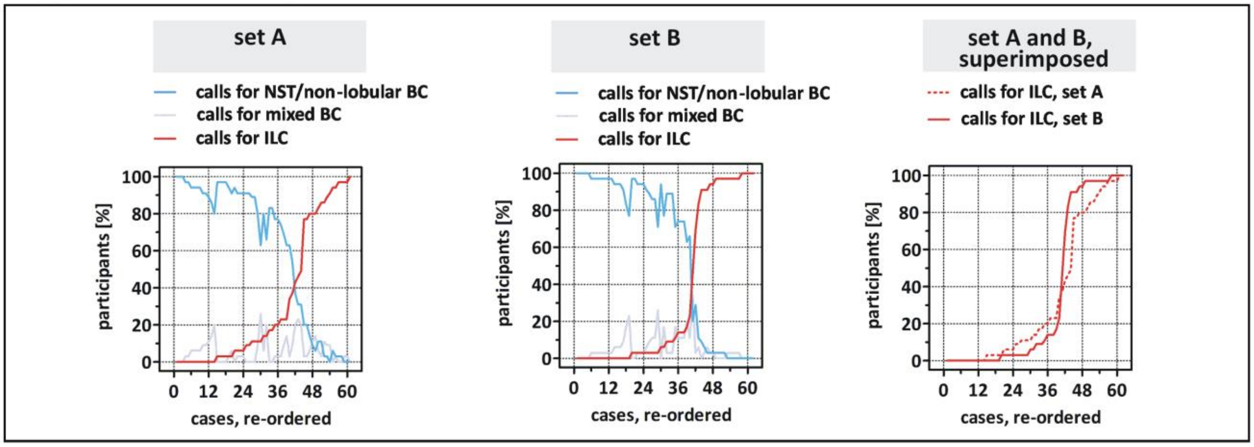

Figure S3. Alternative visualization of BC subtype calls. Cases from set A and set B were re-ordered from left to right, according to increasing participant calls for ILC. Next, percent participant calls for ILC (red), mixed BC NST/ILC (gray), or NST/non-lobular BC was plotted on the y-axis and linked with a connecting line. Please note that in set A just one specimen received 100% participant calls for ILC (upper right corner in the first plot).

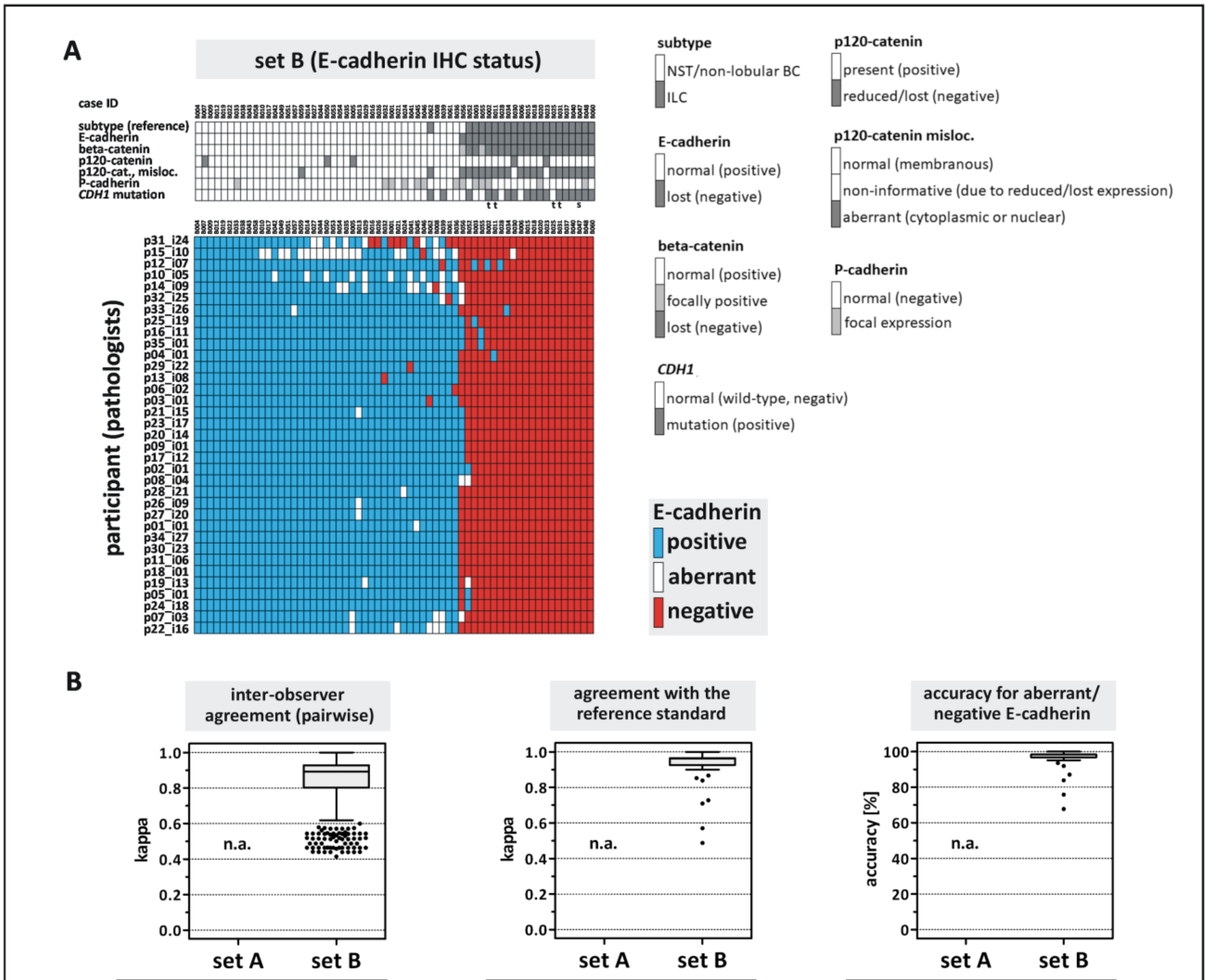

median	n.a.	0.89
IQR	-	0.80-0.93
range	-	0.41-1.00

median	n.a.	0.96
IQR	-	0.93-0.96
range	-	0.49-1.00

median	n.a.	98%
IQR	-	97-98%
range	-	68-100%

Figure S4. E-cadherin status calls. **(A)** Thirty-five experienced pathologists independently classified the E-cadherin IHC status in n=62 BC specimens based on centrally stained sections (set B). Tumor characteristics, as defined by the reference standard are shown in the top panel. A two-dimensional presentation of participant calls is shown in the lower panel. **(B)** Inter-observer agreement (left), agreement with the reference standard for a positive, aberrant or negative E-cadherin status (middle), and agreement with the reference standard for positive or aberrant/negative E-cadherin status (negative and aberrant grouped together) expressed as accuracy [%]. Data are presented as traditional Tukey plots. Horizontal lines indicate the median, boxes indicate the interquartile range (IQR), whiskers indicate the 1.5-fold interquartile distance, or the minimal/maximal values, whichever is shorter.

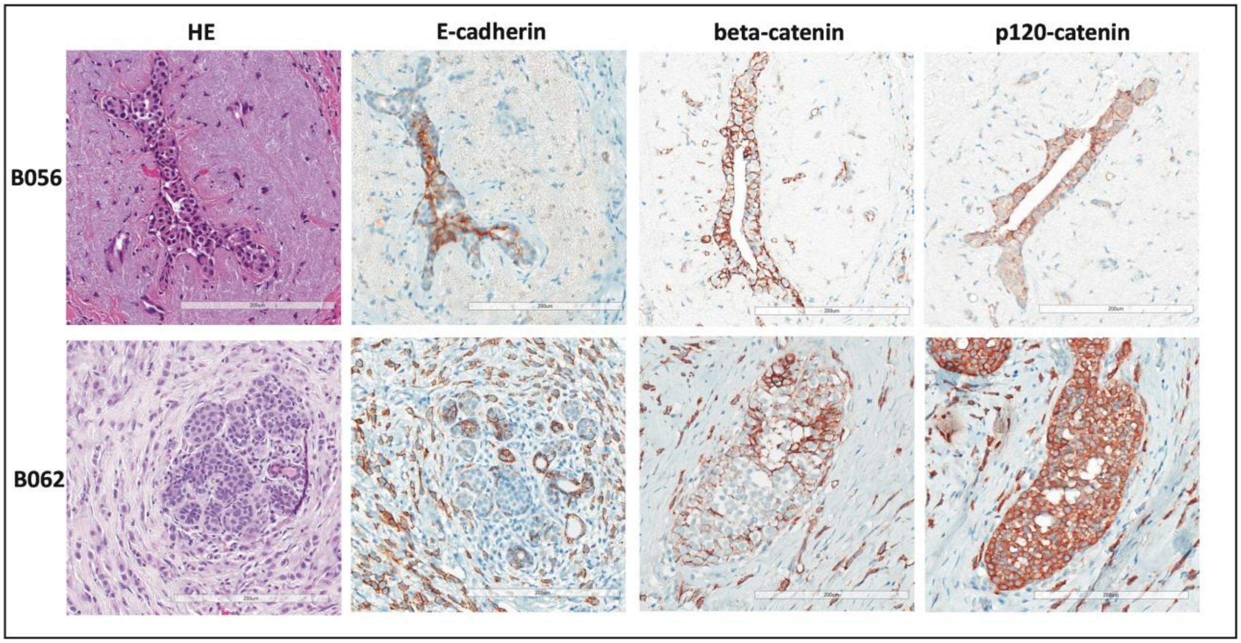

Figure S5. Lobular carcinoma in situ (LCIS) in cases B056 and B062. Representative photomicrographs of HE- and IHC-stained sections at x200 magnification. Scale bars correspond to 200 µm. B056 represents an example of LCIS with pagetoid extension in a mammary duct. LCIS cells (E-cadherin-negative) undermine the luminal epithelial cell layer (E-cadherin-positive). B062 showed the unusual constellation of an E-cadherin-negative LCIS and an E-cadherin-positive ILC with dissociated and single file growth pattern (for details see text). The photomicrographs of the IHC stainings for beta-catenin and p120-catenin were taken from a different LCIS focus, because the lobule depicted in the E-cadherin staining was not represented on the sections of the beta-catenin and p120-catenin staining.

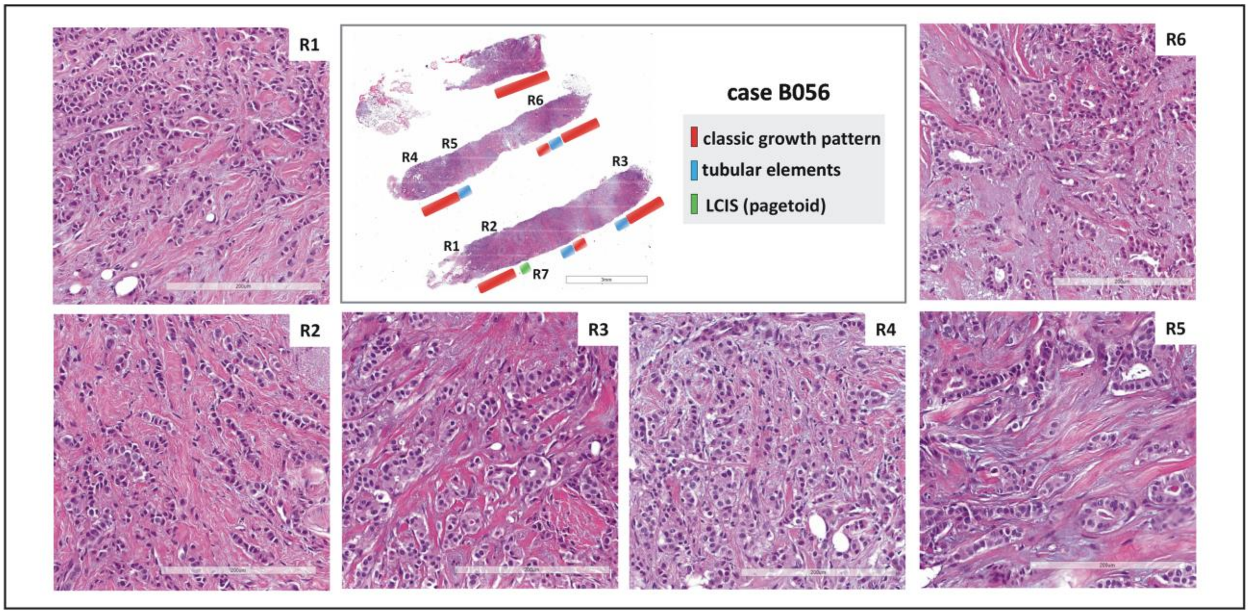

Figure S6. Variable growth pattern in case B056. Representative photomicrographs of HE-stained sections at x5 (center) and x200 (periphery) magnification. Areas with classic growth pattern (single files, dissociated growth) accounted for approximately 80-85%. Areas with tubular elements accounted for approximately 15-20% of the invasive carcinoma. The region with pagetoid LCIS (green, R7) is shown in detail in Supplemental Data Figure 5.

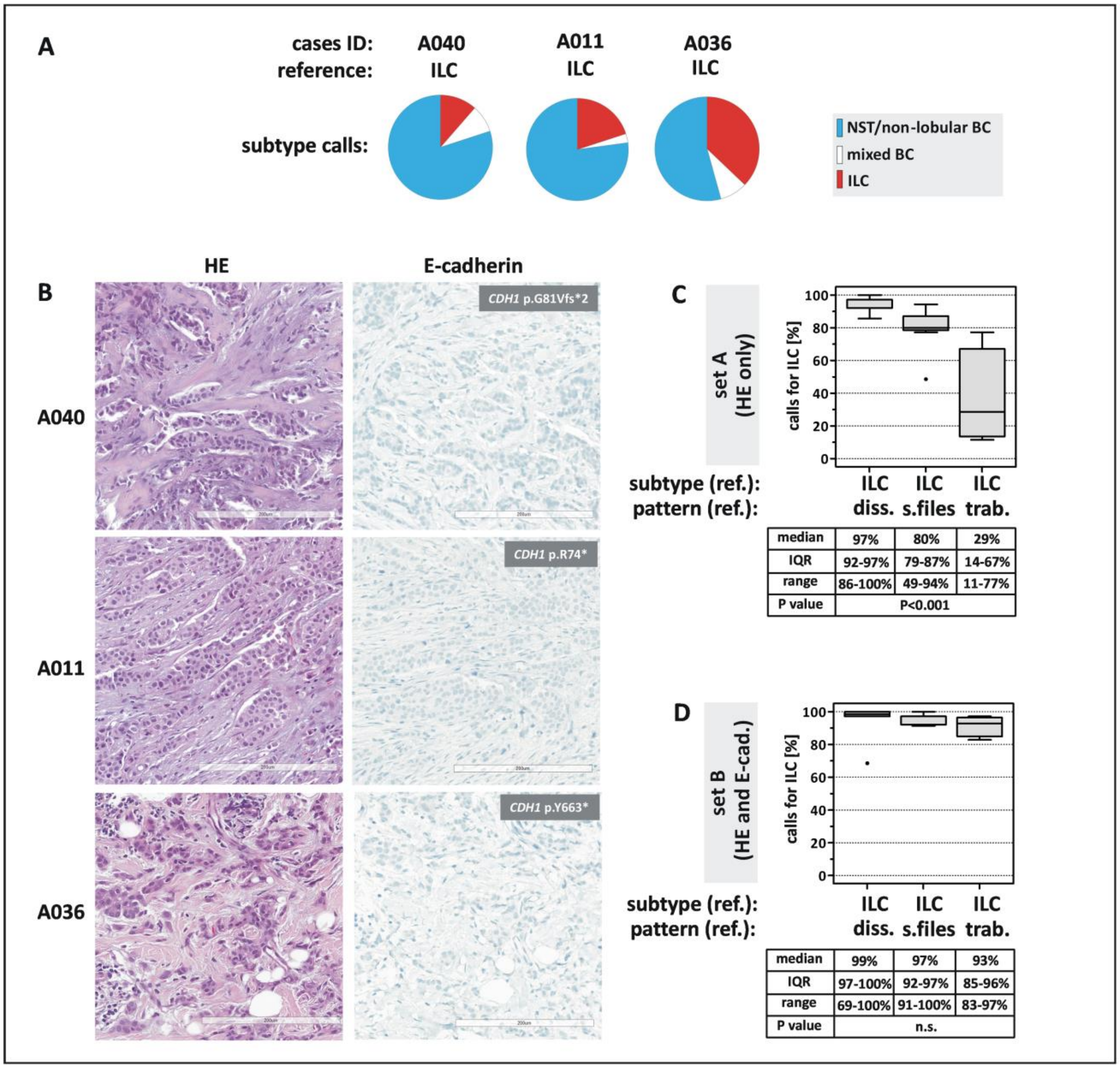

Figure S7. Histology of ILCs classified as NST in set A. **(A)** Pie charts showing proportional subtype calls for cases A040, A011, and A036. Case IDs and BC subtypes according to the reference standard are given on top. **(B)** Representative photomicrographs of HE-stained sections and E-cadherin IHC at x200 magnification. Scale bars correspond to 200 µm. Insets fitted over E-cadherin IHC stainings indicate the *CDH1* mutation status. **(C and D)** Participant calls for ILC in BCs that were classified as ILC with predominantly dissociated growth pattern (diss.), single file growth pattern (s.files), or trabecular growth pattern (trab.) by the reference (ref.). Data are presented as traditional Tukey plots. Horizontal lines indicate the median, boxes indicate the interquartile range (IQR). Statistical significance was determined with the Kruskal-Wallis test.

Table S1. Antibodies and IHC scoring methods

antigen	antibody	source	dilution	antigenic retrieval	detection system	scoring	cutoff
ER	clone SP1	Ventana	undiluted, ready-to use solution	CC1 mild protocol (Ventana)	ultraView DAB Kit (Ventana)	semiquantitative, 0-100	0-5, neg.; 10-100, pos.
PR	clone 1E2	Ventana	undiluted, ready-to use solution	CC1 mild protocol (Ventana)	ultraView DAB Kit (Ventana)	semiquantitative, 0-100	0-5, neg.; 10-100, pos.
HER2	clone 4B5	Ventana	undiluted, ready-to use solution	CC1 mild protocol (Ventana)	ultraView DAB Kit (Ventana)	0, 1+, 2+, 3+	0-1, neg.; 2+, equivocal; 3+, positive
Ki67	clone 30-9	Ventana	undiluted, ready-to use solution	CC1 mild protocol (Ventana)	ultraView DAB Kit (Ventana)	semiquantitative, 0-100	n.a.
CK5/14	XM26+LL002	Diagnostic BioSystems	1:200	CC1 mild protocol (Ventana)	ultraView DAB Kit (Ventana)	i) completely neg.; ii) focally pos. (< approx. 25% cells); iii) positive (> approx. 25% cells)	n.a.
E-cadherin	clone ECH-6	Zytomed	1:100	CC1 mild protocol (Ventana)	ultraView DAB Kit (Ventana)	Remmele IRS	0-1; negative 2-12, positive
beta-catenin	clone 14	BD Transduction Laboratories	1:75	CC1 mild protocol (Ventana)	ultraView DAB Kit (Ventana)	i) completely neg.; ii) focally pos. (< approx. 75% cells); iii) positive (> approx. 75% cells)	n.a.
p120-catenin (expression)	clone 98	BD Transduction Laboratories	1:250	CC1 mild protocol (Ventana)	ultraView DAB Kit (Ventana)	Remmele IRS	0-2, neg.; 3-12, pos.
p120-catenin (misloc.)	clone 98	BD Transduction Laboratories	1:250	CC1 mild protocol (Ventana)	ultraView DAB Kit (Ventana)	i) normal (membranous); ii) aberrant (nuclear or cytosolic); iii) non-informative (low/neg. expression)	n.a.
P-cadherin	clone 56	BD Transduction Laboratories	1:100	CC1 mild protocol (Ventana)	CC1 mild protocol (Ventana)	i) completely neg.; ii) focally pos. (< approx. 75% cells); iii) positive (> approx. 75% cells)	n.a.

Table S2. Tumor characteristics of NST/non-lobular BC, as defined by the reference standard, are balanced between set A and B.

	reference	all cases		set A		set B			
		n	%	n	%	n	%	test	P value
all NST/nonlobular BC cases		81	100	40	49	41	51		
grade	G1	3	100	1	33	2	67	CSTT	0.459
	G2	47	100	26	55	21	45		
	G3	31	100	13	42	18	58		
mBSR: architecture	1	0	0	0	0	0	0	CSTT	0.286
	2	22	100	13	59	9	41		
	3	59	100	27	46	32	54		
mBSR: nuc. grade	1	0	0	0	0	0	0	CSTT	0.904
	2	44	100	22	50	22	50		
	3	37	100	18	49	19	51		
mBSR: proliferation	1	12	100	8	67	4	33	CSTT	0.357
	2	52	100	24	46	28	54		
	3	17	100	8	47	9	53		
ER	neg.	1	100	1	100	0	0	FET	0.488
	pos.	79	100	38	48	41	52		
	n.a.	1	100	1	100	0	0		
PR	neg.	7	100	3	43	4	57	FET	1.000
	pos.	74	100	37	50	37	50		
HER2	0/1+	62	100	29	47	33	53	CSTT	0.576
	2+/F.-neg.	16	100	10	63	6	37		
	2+/F. n.a.	2	100	1	50	1	50		
	3+, 2+/F.-pos.	1	100	0	0	1	100		
Ki67	<10%	4	100	3	75	1	25	CSTT	0.725
	10-19%	19	100	8	42	11	58		
	20-34%	49	100	25	51	24	49		
	35-100%	9	100	4	44	5	56		
E-cadherin	neg.	1	100	0	0	1	100	FET	1.000
	aberrant	0	0	0	0	0	0		
	pos.	80	100	40	50	40	50		
CDH1 status	wild-type	80	100	40	50	40	50	FET	1.000
	mutant	1	100	0	0	1	100		
beta-catenin	neg.	0	0	0	0	0	0	CSTT	0.320
	focally pos.	1	100	0	0	1	100		
	pos.	80	100	40	50	40	50		
p120-catenin	low/neg.	4	100	1	25	3	75	FET	0.616
	pos.	77	100	39	51	38	49		
p120-catenin mislocation	membranous	75	100	39	52	36	48	FET	0.240
	mislocated	2	100	0	0	2	100		
	not informative	4	100	1	25	3	75		
P-cadherin	neg.	67	100	34	51	33	49	FET	0.770
	focally pos.	14	100	6	43	8	57		
	pos.	0	0	0	0	0	0		

FET; Fisher's exact test (set A *versus* set B), CSTT; Chi square test for trends (set A *versus* set B), pos.; positive, neg.; negative.

Table S3. Tumor characteristics of ILCs, as defined by the reference standard, are balanced between set A and B

	reference	all cases		set A		set B			
		n	%	n	%	n	%	test	P value
all ILC cases		42	100	21	50	21	50		
grade	G1	1	100	0	0	1	100	CSTT	0.740
	G2	31	100	17	55	14	45		
	G3	10	100	4	40	6	60		
mBSR: architecture	1	0	0	0	0	0	0		n.a.
	2	0	0	0	0	0	0		
	3	42	100	21	50	21	50		
mBSR: nuc. grade	1	7	100	3	43	4	57	CSTT	0.781
	2	29	100	16	55	13	45		
	3	6	100	2	33	4	67		
mBSR: proliferation	1	10	100	5	50	5	50	CSTT	0.464
	2	25	100	14	56	11	44		
	3	7	100	2	29	5	71		
ER	neg.	0	0	0	0	0	0		n.a.
	pos.	42	100	21	50	21	50		
	n.a.	0	0	0	0	0	0		
PR	neg.	3	100	2	67	1	33	FET	1.000
	pos.	39	100	19	49	20	51		
HER2	0/1+	38	100	18	47	20	53	CSTT	0.293
	2+/F.-neg.	4	100	3	75	1	25		
	2+/F. n.a.	0	0	0	0	0	0		
	3+, 2+/F.-pos.	0	0	0	0	0	0		
Ki67	<10%	2	100	0	0	2	100	CSTT	0.460
	10-19%	18	100	9	50	9	50		
	20-34%	21	100	12	57	9	43		
	35-100%	1	100	0	0	1	100		
E-cadherin	neg.	40	100	20	50	20	50	FET	1.000
	aberrant	0	0	0	0	0	0		
	pos.	2	100	1	50	1	50		
CDH1 status	wild-type	10	100	5	50	5	50	FET	1.000
	mutant	32	100	16	50	16	50		
beta-catenin	neg.	37	100	18	49	19	51	CSTT	0.750
	focally pos.	3	100	2	67	1	33		
	pos.	2	100	1	50	1	50		
p120-catenin	low/neg.	8	100	6	75	2	25	FET	0.238
	pos.	34	100	15	44	19	56		
p120-catenin mislocation	membranous	1	100	1	100	0	0	FET	0.441
	mislocated	33	100	14	42	19	58		
	not informative	8	100	6	75	2	25		
P-cadherin	neg.	35	100	19	54	16	46	FET	0.410
	focally pos.	7	100	2	29	5	71		
	pos.	0	0	0	0	0	0		
variant/growth pattern$	classical/dissociated	17	100	9	53	8	47	FET*	1.00
	classical/single files	16	100	8	50	8	50		
	trabecular	8	100	4	50	4	50		
	solid	1	100	0	0	1	100		
	pleo., alv., others	0	0	0	0	0	0		

FET; Fisher's exact test (set A *versus* set B), CSTT; Chi square test for trends (set A *versus* set B), pos.; positive, neg.; negative. Pleo.; pleomorphic, alv.; alveolar, others refers to histiocytoid ILC and other rare variants. *Classical versus non-classical. $Refers to the predominant growth pattern.

Table S4. ILCs, as defined by the reference standard, are associated with loss of E-cadherin expression and *CDH1* mutation

	reference	all cases (n=123)					set A (n=61)					set B (n=62)					
		NST		ILC			NST		ILC			NST		ILC			
		n	%	n	%	P value	n	%	n	%	P value	n	%	n	%	P value	test
all cases		81	100	42	100		40	49	21	50		41	51	21	50		
grade	G1	3	4	1	2	0.188	1	2	0	0	0.393	2	5	1	5	0.317	CSTT
	G2	47	58	31	74		26	65	17	81		21	51	14	67		
	G3	31	38	10	24		13	33	4	19		18	44	6	28		
mBSR: architecture	1	0	0	0	0	P<0.001	0	0	0	0	0.003	0	0	0	0	0.020	CSTT
	2	22	27	0	0		13	32	0	0		9	22	0	0		
	3	59	73	42	100		27	68	21	100		32	78	21	100		
mBSR: nuc. grade	1	0	0	7	17	P<0.001	0	0	3	14	0.001	0	0	4	19	0.003	CSTT
	2	44	54	29	69		22	55	16	76		22	54	13	62		
	3	37	46	6	14		18	45	2	10		19	46	4	19		
mBSR: proliferation	1	12	15	10	24	0.252	8	20	5	24	0.386	4	10	5	24	0.452	CSTT
	2	52	64	25	59		24	60	14	67		28	68	11	52		
	3	17	21	7	17		8	20	2	9		9	22	5	24		
ER	neg.	1	1	0	0	1.000	1	2	0	0	1.000	0	0	0	0	-	FET
	pos.	79	98	42	100		38	95	21	100		41	100	21	100		
	n.a.	1	1	0	0		1	3	0	0		0	0	0	0		
PR	neg.	7	9	3	7	1.000	3	7	2	10	1.000	4	10	1	5	0.654	FET
	pos.	74	91	39	93		37	93	19	90		37	90	20	95		
HER2	0/1+	62	77	38	90	0.088	29	73	18	86	0.309	33	81	20	95	0.156	CSTT
	2+/F.-neg.	16	20	4	10		10	25	3	14		6	15	1	5		
	2+/F. n.a.	2	2	0	0		1	2	0	0		1	2	0	0		
	3+, 2+/F.-pos.	1	1	0	0		0	0	0	0		1	2	0	0		
Ki67	<10%	4	5	2	5	0.034	3	7	0	0	0.320	1	2	2	9	0.049	CSTT
	10-19%	19	23	18	43		8	20	9	43		11	27	9	43		
	20-34%	49	61	21	50		25	63	12	57		24	59	9	43		
	35-100%	9	11	1	2		4	10	0	0		5	12	1	5		
E-cadherin	neg.	1	1	40	95	P<0.001	0	0	20	95	P<0.001	1	2	20	95	P<0.001	FET
	aberrant	0	0	0	0		0	0	0	0		0	0	0	0		
	pos.	80	99	2	5		40	100	1	5		40	98	1	5		
CDH1 status	wild-type	80	99	10	24	P<0.001	40	100	5	24	P<0.001	40	98	5	24	P<0.001	FET
	mutant	1	1	32	76		0	0	16	76		1	2	16	76		
beta-catenin	neg.	0	0	37	88	P<0.001	0	0	18	86	P<0.001	0	0	19	90	P<0.001	CSTT
	focally pos.	1	1	3	7		0	0	2	9		1	2	1	5		
	pos.	80	99	2	5		40	100	1	5		40	98	1	5		
p120-catenin	neg.	4	5	8	19	0.022	1	3	6	29	0.005	3	7	2	10	1.000	FET
	pos.	77	95	34	81		39	98	15	71		38	93	19	90		
p120-catenin mislocation	membranous	75	93	1	2	P<0.001	39	98	1	5	P<0.001	36	88	0	0	P<0.001	FET
	mislocated	2	2	33	79		0	0	14	67		2	5	19	90		
	not informative	4	5	8	19		1	2	6	28		3	7	2	10		
P-cadherin	neg.	67	83	35	83	1.000	34	85	19	90	0.703	33	80	16	76	0.748	FET
	focally pos.	14	17	7	17		6	15	2	10		8	20	5	24		
	pos.	0	0	0	0		0	0	0	0		0	0	0	0		

FET; Fisher's exact test, CSTT; Chi square test for trends, pos.; positive, neg.; negative.

Table S5. Proportions of category II cases in set A and set B

calls for ILC		explanation	category	all cases (n=123)		set A (n=61)		set B (n=62)	
n	%			n	%	n	%	n	%
0/35	0	**complete consensus against ILC**	cat. I	55	45	24	39	31	50
1/35	3	one dissenting call for ILC							
2/35	6	two dissenting calls for ILC							
3/35	9	three dissenting calls for ILC	cat. II	45	36	30	49	15	24
18/35	51	balanced calls for/against ILC							
32/35	91	three dissenting calls against ILC							
33/35	94	two dissenting calls against ILC	cat. III	23	19	7	12	16	26
34/35	97	one dissenting call against ILC							
35/35	100	**complete consensus for ILC**							
			test	FET (cat. I+III vs cat. II)					
			P value	P=0.005					

FET; Fisher's exact test.

Table S6. Participants' written comments for case B056.

participant	comment
p01_i01	LIN present. ILC-like but tubules.
p02_i01	Possibly tubulo-lobular?
p04_i01	ILC but tubular too.
p07_i03	Heterogeneous.
p08_i04	E-cadherin seems reliably negative.
p09_i01	Difficult. I'm undecided.
p11_i06	Partially tubules.
p14_i09	Not easy. Impression of a lobular component.
p14_i09	Is E-cad. False-negative in the ductal component?
p18_i01	LIN present. Partially lobular and ductal pattern.<br>What are the molecular features?
p21_i15	Some areas show a delicate and faint E-cad.-positivity.<br>Surprisingly low E-cad. staining in well differentiated glands. I'm in favor of a mixed NST+lobular or tubulo-lobular BC.
p22_i16	Considered as ILC (although there are some tubules).
p23_i17	Tubular-lobular growth. E-cad.: NST area with very weak expression. ILC area shows a complete loss.
p24_i18	Some cells are possibly very weakly E-cadherin-positive?
p27_i20	I'm not sure: In some areas it is unequivocally lobular.<br>In other areas the growth pattern would correspond to NST.<br>E-cad. didn't help me to decide.
p32_i25	I don't want to believe E-cad., but it looks real.<br>I would sign out as carcinoma with mixed features.
p33_i26	Very weak focal expression of E-cad. in tubular structures?

Table S7. Features of BCs with variable subtype calls (category II specimens)

	reference	all cases (n=123)							set A (n=61)							set B (n=62)							
		cat. I		cat. II		cat. III			cat. I		cat. II		cat, III			cat. I		cat. II		cat. III			
		n	%	n	%	n	%	P value	n	%	n	%	n	%	P value	n	%	n	%	n	%	P value	test
all cases		55	100	45	100	23	100		24	100	30	100	7	100		31	100	15	100	16	100		
grade	G1	3	6	0	0	0	0	0.336	1	4	0	0	0	0	0.797	2	7	0	0	0	0	0.451	CST
	G2	32	58	31	69	16	70		17	71	21	70	5	71		15	48	10	67	11	69		
	G3	20	36	14	31	7	30		6	25	9	30	2	29		14	45	5	33	5	31		
mBSR: architecture	1	0	0	0	0	0	0	<0.001	0	0	0	0	0	0	<0.001	0	0	0	0	0	0	0.019	CST
	2	21	38	2	4	0	0		12	50	1	3	0	0		9	29	1	7	0	0		
	3	34	62	43	96	23	100		12	50	29	97	7	100		22	71	14	93	16	100		
mBSR: nuc. grade	1	0	0	2	5	5	22	<0.001	0	0	2	7	1	14	0.386	0	0	0	0	4	25	0.002	CST
	2	29	53	29	64	15	65		14	58	19	63	5	71		15	48	10	67	10	63		
	3	26	47	14	31	3	13		10	42	9	30	1	14		16	52	5	33	2	13		
mBSR: proliferation	1	9	16	10	22	3	13	0.668	5	21	8	27	0	0	0.12	4	13	2	13	3	19	0.937	CST
	2	33	60	29	65	15	65		12	50	20	67	6	86		21	68	9	60	9	56		
	3	13	24	6	13	5	22		7	29	2	6	1	14		6	19	4	27	4	25		
ER	neg.	0	0	1	2	0	0	0.474	0	0	1	3	0	0	0.711	0	0	0	0	0	0	0.999	CST
	pos.	55	100	43	96	23	100		24	100	28	94	7	100		31	100	15	100	16	100		
	n.a.	0	0	1	2	0	0		0	0	1	3	0	0		0	0	0	0	0	0		
PR	neg.	4	7	2	4	3	13	0.436	2	8	1	3	2	29	0.091	2	6	1	7	1	6	0.999	CST
	pos.	51	93	43	96	20	87		22	92	29	97	5	71		29	94	14	93	15	94		
HER2	0/1+	43	78	37	82	21	91	0.702	19	79	23	77	6	86	1,000	24	78	14	93	15	94	0.709	CST
	2+/F.-neg.	10	18	8	18	2	9		5	21	7	23	1	14		5	16	1	7	1	6		
	2+/F. n.a.	1	2	0	0	0	0		0	0	0	0	0	0		1	3	0	0	0	0		
	3+, 2+/F.-pos.	1	2	0	0	0	0		0	0	0	0	0	0		1	3	0	0	0	0		
Ki67	<10%	4	7	2	4	0	0	0.445	3	13	0	0	0	0	0.154	1	3	2	13	0	0	0.357	CST
	10-19%	12	22	15	33	10	43		3	13	12	40	2	29		9	29	3	20	8	50		
	20-34%	33	60	25	56	12	52		16	67	16	53	5	71		17	55	9	60	7	44		
	35-100%	6	11	3	7	1	4		2	8	2	7	0	0		4	13	1	7	1	6		
E-cadherin	neg.	0	0	18	40	23	100	<0.001	0	0	13	43	7	100	<0.001	0	0	5	33	16	100	<0.001	CST
	aberrant	0	0	0	0	0	0		0	0	0	0	0	0		0	0	0	0	0	0		
	pos.	55	100	27	60	0	0		24	100	17	57	0	0		31	100	10	67	0	0		
CDH1 status	wild-type	55	100	30	67	5	22	<0.001	24	100	19	63	2	29	<0.001	31	100	11	73	3	19	<0.001	CST
	mutant	0	0	15	33	18	78		0	0	11	37	5	71		0	0	4	27	13	81		
beta-catenin	neg.	0	0	14	31	23	100	<0.001	0	0	11	36	7	100	<0.001	0	0	3	20	16	100	<0.001	CST
	focally pos.	0	0	4	9	0	0		0	0	2	7	0	0		0	0	2	13	0	0		
	pos.	55	100	27	60	0	0		24	100	17	57	0	0		31	100	10	67	0	0		
p120-catenin	neg.	4	7	3	7	5	22	0.099	1	4	3	10	3	43	0.017	3	10	0	0	2	12	0.397	CST
	pos.	51	93	42	93	18	78		23	96	27	90	4	57		28	90	15	100	14	88		
p120-catenin misloc.	membranous	50	91	26	58	0	0	<0.001	23	96	17	57	0	0	<0.001	27	87	9	60	0	0	<0.001	CST
	mislocated	1	2	16	36	18	78		0	0	10	33	4	57		1	3	6	40	14	88		
	not informative	4	7	3	6	5	22		1	4	3	10	3	43		3	10	0	0	2	12		
P-cadherin	neg.	49	89	34	76	19	83	0.201	21	88	26	87	6	86	0.991	28	90	8	53	13	81	0.015	CST
	focally pos.	6	11	11	24	4	17		3	12	4	13	1	14		3	10	7	47	3	19		
	pos.	0	0	0	0	0	0		0	0	0	0	0	0		0	0	0	0	0	0		
E- / P-cadherin	neg. / neg.	0	0	14	31	19	83	<0.001	0	0	12	40	6	86	<0.001	0	0	2	13	13	81	<0.001	CST
	neg. / focally pos.	0	0	4	9	4	17		0	0	1	3	1	14		0	0	3	20	3	19		
	pos. / neg.	49	89	20	44	0	0		21	88	14	47	0	0		28	90	6	40	0	0		
	pos. / focally pos.	6	11	7	16	0	0		3	12	3	10	0	0		3	10	4	27	0	0		

CST; Chi square test for trends. Pos.; positive, neg.; negative.
*Cutoffs to separate category I, II, and III were set at <3/35 (<9%) and >32/35 (>91%) participant calls for ILC.

Table S8. Educational BC subtype assessment*

	inter-observer agreement (pairwise)		agreement with the reference standard			accuracy for detection of a lobular component	
	set A	set B	set A	set B		set A	set B
median [kappa]	0.36	0.35	0.38	0.55	median [%]	75%	77%
IQR	0.26-0.44	0.22-0.47	0.30-0.51	0.25-0.69	IQR	67-80%	62-90%
range	0.06-0.67	-0.01-0.87	0.10-0.70	0.10-0.90	range	51-85%	52-95%
P value	n.s.		P=0.015			n.s.	

*Participants included 15 medical students, 1 pathologist assistant educated in histopathology, 1 early resident in clinical pathology, and 1 biomedical scientists. All medical students had attended a course in basic histopathology at the MHH (40 hours lectures, 40 hours practical training in microscopy using 122 different tissue specimens, including 1 representative ductal carcinoma in situ, 1 representative invasive BC of NST, and 1 representative ILC) before participation.
